# Polyaniline-hybridized quantum-confined 1D lepidocrocite titania for ultra-high-energy supercapacitors

**DOI:** 10.1039/d6ta05686e

**Published:** 2026-11-09

**Authors:** Sukanya Maity, Joseph Halim, Ali Saffar Shamshirgar, Risha Achaiah Iythichanda, Per O. Å. Persson, Michel W. Barsoum, Johanna Rosen

**Affiliations:** a Department of Physics, Chemistry and Biology (IFM), Linköping University 58183 Linköping Sweden sukanya.maity@liu.se johanna.rosen@liu.se; b Department of Materials Science and Engineering, Drexel University 19104 Pennsylvania Philadelphia USA barsoumw@drexel.edu

## Abstract

High-energy capacitors have garnered significant interest due to their dual ability to store energy and deliver high power, influenced by the properties of their electrode materials. Herein, we report a unique composite structure created through *in situ* polymerization of polyaniline (PANI) in the presence of one-dimensional lepidocrocite (1DL) titania nanofilaments (NFs). The hierarchical 1DL NF structures allow direct and fast ion transport, enhancing both capacity and stability when combined with PANI. The resultant composite (PANI : 1DLs in a 2 : 1 wt ratio) exhibits a specific capacity of 377 mA h g^−1^ at 0.5 Ag^−1^, with high mass loading (∼5 mg cm^−2^). The composite's performance in a symmetric cell configuration demonstrates a specific energy of 154 Wh kg^−1^ at 0.5 A g^−1^, which is the highest specific energy among other reported TiO_2_-based supercapacitor electrodes over a wide voltage window of 3.5 V. A peak specific power of 17 kW kg^−1^ was measured while maintaining a specific energy of 31Wh kg^−1^. After 10 000 charge–discharge cycles, the cell retained ∼56% of its initial capacity with 94% coulombic efficiency. To enhance cell stability and reduce self-discharge, an asymmetric arrangement with activated carbon improved voltage retention, resulting in a 29.7% open-circuit potential retention after 24 h and 80% capacitance retention after post-cycling. The synthesis method is inexpensive, sustainable, and potentially scalable, highlighting the potential for high-energy supercapacitors.

## Abstract 2

Polyaniline hybridized with 1D lepidocrocite TiO_2_ enhances ion and electron transport. This composite offers high charge-storage capacity for supercapacitors in organic electrolytes, demonstrating its potential for advanced energy storage.

## Graphical abstract

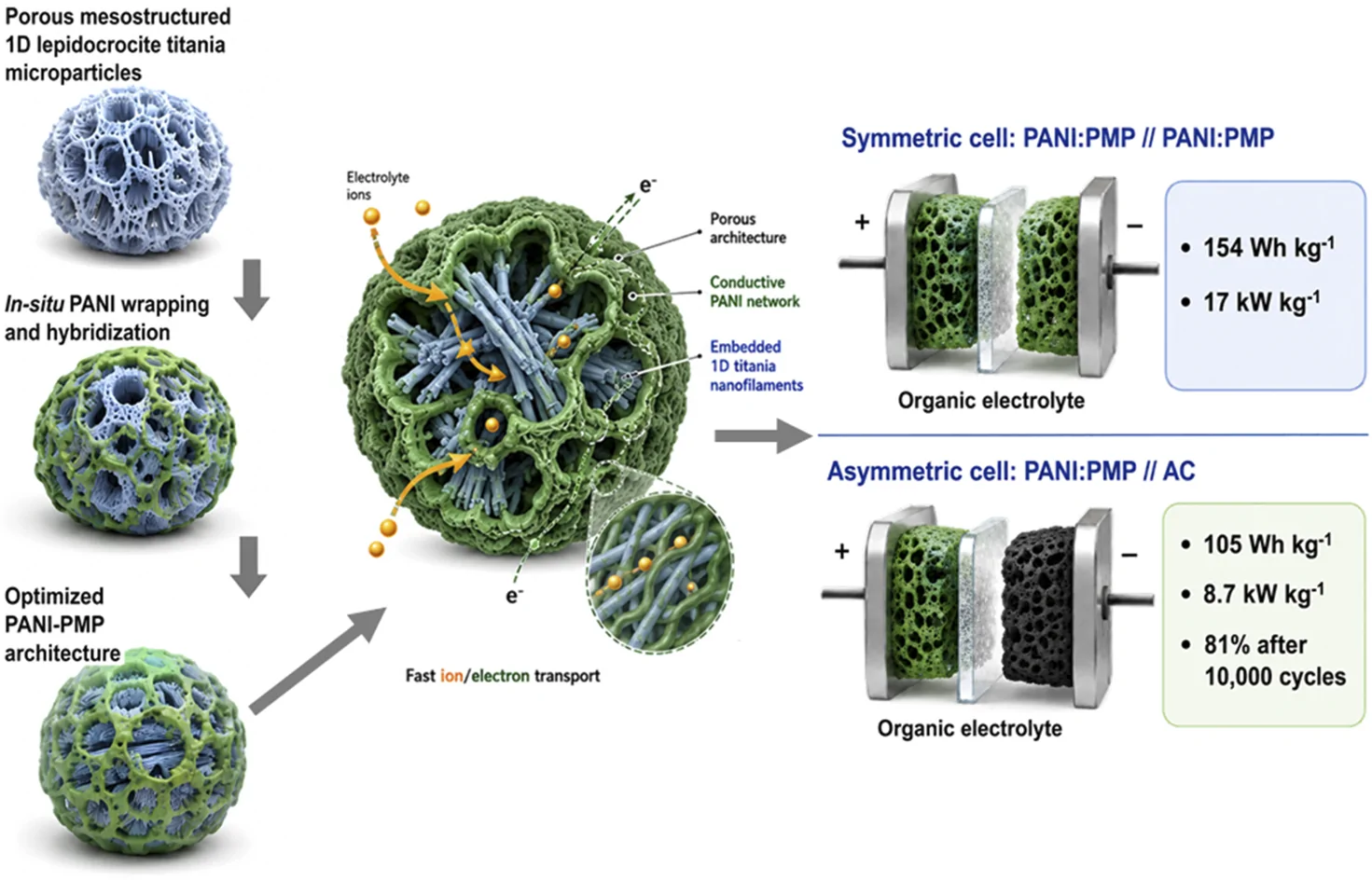

## Introduction

Advancements in cutting-edge technology for intelligent and portable electronics, renewable energy integration, automotive, and other industrial systems are driving the demand for high-performance energy storage devices.^1,2^ Supercapacitors (SCs) are a sustainable and efficient electrochemical energy storage system with significant practical value, as they can deliver high power densities, rapid charging and discharging, and superior cycling capabilities. The choice of electrode materials plays the most important role in the charge storage mechanism.^3^ Designing a state-of-the-art SC electrode that can store high energy and power efficiently by utilizing the properties of batteries and capacitors in a single system is critical for developing useful energy systems.^4^

One-dimensional titania (TiO_2_) nanostructures possess unique properties and versatile characteristics. Their unique architecture and morphologies expand their applications to photo/electro-catalysis, photoluminescence, environmental remediation and energy storage.^5^ We recently developed a straightforward, bottom-up, one-pot synthesis method for TiO_2_-based 1D NFs by reacting Ti-containing precursors (*e.g.* TiB_2_, TiC, TiOSO_4_) with tetraalkylammonium, TAA, hydroxide solutions at temperatures < 100 °C under atmospheric pressure.^6,7^ The resulting lepidocrocite-based NFs will be referred to as 1DLs. These NFs display various morphologies depending on the solvent used in post-processing. When ethanol (EtOH) – used to precipitate the 1DL and reduce the pH of the product mixture and remove excess TAA – evaporates, the 1DLs self-assemble into porous mesostructured particles (PMPs). The PMPs are almost spherical in shape, with diameters of a few microns, resulting in a free-flowing powder.^8–10^ Replacing EtOH with water, however, creates a stable, colloid that, when filtered, results in self-standing films of pseudo-2D flakes composed of overlapping fibrils.

In our initial discovery and until quite recently, more than a dozen Ti precursors were water-insoluble.^6–10^ Recently, we discovered a facile, more economical, and potentially scalable synthesis of 1DL NFs using a water-soluble Ti precursor, *viz.* titanium oxysulfate, TiOSO_4_. This precursor allows for a faster and less expensive process, overcoming the limitations of our previous methods.^11^

Quantum confinement in the 1DL structure arises from the strong spatial restriction of charge carriers as the lepidocrocite structure is reduced from a two-dimensional, 2D, sheet to an ultranarrow 1D ribbon. Experimentally, Walter *et al.*^12^ demonstrated that the band gap energy, *E*_g_, of dried 1DL films can be tuned from approximately 3.5 to 4.5 eV by decreasing the concentration of the precursor colloidal suspension from 40 to 0.01 g L^−1^; dilute suspensions favor more isolated and narrower filaments after drying, whereas concentrated suspensions promote lateral self-assembly into wider ribbons or sheet-like structures, progressively weakening the confinement effect. First-principles calculations further support this interpretation. Liu *et al.*^13^ showed that water-terminated 1DL structures with widths ranging from approximately 15.1 Å (4C) to only 4.9 Å (1C) exhibit increasingly strong confinement, with the narrowest 1C structure reaching a calculated band gap of about 4.5 eV, in excellent agreement with experimentally observed values.

In terms of applications relevant to this work, TiO_2_ is a thoroughly researched electrode material for supercapacitors, SCs, in various morphologies (including 0-D nanoparticles, 1-D nanowires, nanorods, nanofibers, nanotubes, 2-D nanosheets, and 3-D nanoparticles)^14,15^ and composite forms (with carbon-based materials, metal ion doping, and the integration of pseudocapacitive materials).^16–18^ Additionally, black titanium oxide (B-TiO_*x*_), named for its black appearance, has also been explored as an electrode for SCs due to its comparatively improved electrical conductivity.^19^ However, the various structural controls aimed at enhancing electrochemical performance did not lead to high-energy (>10^2^ Wh kg^−1^) storage systems.

SC electrodes rely on both ionic and electronic conduction. Conducting polymers such as polyaniline (PANI) are well-investigated for SC electrodes, as they provide high capacitance, enhance the overall electronic conductivity, are inexpensive and easy to synthesize, and have tunable properties.^20–22^ However, because they store charge by doping/undoping on the polymer backbone, they suffer from poor mechanical and cycle stability.^23–25^ In the past decade, several researchers have tried to incorporate TiO_2_ with PANI for improved SC electrodes, and a summary is presented in Table 1. Despite these efforts, achieving ultrahigh energy density has remained a challenge. Recently, we discovered that when TiB_2_-derived Li-intercalated 1DLs are mixed with activated carbon (AC), excellent capacities (1024 F g^−1^) and energy densities (54 Wh kg^−1^) that are stable for ∼100 000 charge–discharge cycles are obtained.^26^

**Table 1:** PANI and TiO_2_-based composites for SC electrodes. Subtraction of the ambient spectrum was deemed unnecessary, as residual CO_2_ and H_2_O signals were negligible

Electrode	Electrolyte	Configuration	Specific capacity/capacitance	Energy density	Power density	References
PANI@TiO_2_–CuO	1 M HCl	3-Electrode	296 mF cm^−2^ at 1 mA cm^−2^	—	—	27
PANI/sulfonated graphene oxide/TiO_2_ nanorods	1 M H_2_SO_4_	2-Electrode	460 F g^−1^ at 0.7 A g^−1^	23 Wh kg^−1^ at 0.7 A g^−1^	4000 W kg^−1^ at 7 A g^−1^	28
PANI–TiO_2_	1 M H_2_SO_4_	3-Electrode	776 F g^−1^ at 5 mV s^−1^	174 Wh kg^−1^ at 0.5 mA cm^−2^	892 W kg^−1^ at 0.5 mA cm^−2^	29
PANI/Ti sheet	1 M H_2_SO_4_	3-Electrode	1174 mF cm^−2^ at 2 mA cm^−2^	3.05 mWh cm^−2^ at 2 mA cm^−2^	2199 mWh cm^−2^ at 10 mA cm^−2^	30
PANI/CNT/TiO_2_	0.5 M H_2_SO_4_	3-Electrode	477.1 F g^−1^ at 0.4 µA mm^−2^	—	—	31
PANI/TiO_2_/FTO	1 M H_2_SO_4_	3-Electrode	781 F g^−1^ at 0.7 A g^−1^	—	—	32
FTO/TiO_2_/CdS-QDs/PANI	1 M PVP/KOH	2-Electrode	96.7 F g^−1^ at 0.5 A g^−1^	32.4 Wh kg^−1^ at 0.5 A g^−1^	—	33
Polyacrylonitrile–polyphenyl/TiO_2_	1 M KOH	3-Electrode	484 F g^−1^ at 5 mA g^−1^	16.8 Wh kg^−1^ at 5 mA g^−1^	2749.1 W kg^−1^ at 5 mA g^−1^	34
Polyaniline/black TiO_2_ nanotubes	1 M HCl	3-Electrode	999 F g^−1^ at 0.6 A g^−1^	110 Wh kg^−1^, 1.2 A g^−1^	740.55 W kg^−1^ 1.2 A g^−1^	35
rGO/TiO_2_/PANI	1 M H_2_SO_4_	2-Electrode	666.53 F g^−1^ at 0.02 A g^−1^	7.79 Wh kg^−1^ at 4 A g^−1^	8910 W kg^−1^ at 10 A g^−1^	36
PANI/TiO_2_ nanotube arrays	0.5 M Na_2_SO_4_	3-Electrode	512.4 mF cm^−2^	—	—	37
TiO_2_/PVA/PANI	6 M KOH	3-Electrode	140.04 F g^−1^ at 0.5 A g^−1^	16.76 Wh kg^−1^ at 0.5 A g^−1^	938.88 at 2 A g^−1^	38
PA/self-doped TiO_2_ nanotubes	1.0 M HCl	3-Electrode	1084 F g^−1^ at 0.6 A g^−1^	—	—	39
TiO_2_–C nanowire arrays@PANI	1 M H_2_SO_4_	3-Electrode	1818 F g^−1^ at a current density of 1 A g^−1^	—	—	40
PANI/nTiO_2_	0.1 M H_2_SO_4_	2-Electrode	813 F g^−1^ at 1 mA cm^−2^	16.8 Wh kg^−1^ at 1 mA cm^−2^	9.75 W kg^−1^ at 5 mA cm^−1^	41
PANI/PVA/TiO_2_	H_2_SO_4_/PVA	2-Electrode	127.5 F g^−1^ at 0.5 A g^−1^	2.8 Wh kg^−1^ at 0.5 A g^−1^	—	42
PANI coated RuO_2_/TiO_2_	0.1 M H_2_SO_4_	2-Electrode	67.4 F g^−1^ at 0.2 A g^−1^	3.37 Wh kg^−1^ at 0.2 A g^−1^	60 W kg^−1^ at 0.2 A g^−1^	43
PANI/AC/TiO_2_	1.0 M LiClO_4_ in propylene carbonate	3-Electrode	286 F g^−1^	—	—	44
PANI–PMP	1 M TEABF_4_ in acetonitrile	2-Electrode	290 C g^−1^ (∼145 F g^−1^) at 0.5 A g^−1^	154 Wh kg^−1^ at 0.5 A g^−1^	17 kW kg^−1^ at 5 mA g^−1^	This work

This work discusses an exclusive composite structure made by *in situ* wrapping (Fig. 1A) of TiOSO_4_-derived PMPs composed of 1DL NFs (Fig. 1B) with PANI to create improved SC electrode materials. The method is simple, one-pot, cost-effective, and potentially scalable. Moreover, the composite consists of earth-abundant elements, enhancing its sustainability. The synergistic PANI–PMP effect of their composites results in excellent electrochemical behavior. The near-surface redox reactions (from 1DL NFs) and pseudocapacitive counter ion interactions (from PANI) present an intermediate performance between capacitors (power density > 10^4^ W kg^−1^) and batteries (energy density > 10^2^ Wh kg^−1^). Since PANI degrades over time in highly acidic or alkaline environments, we chose an organic electrolyte, specifically tetraethylammonium tetrafluoroborate, TEABF_4_, in an acetonitrile solution. This electrolyte choice expands the SC's operating potential to 3.5 V, double that of the aqueous electrolytes. This is one of the reasons our symmetric SC cell exhibits the highest specific energy (154 Wh kg^−1^) compared to other reported TiO_2_-based SC electrode materials (Table 1).

**Fig. 1:**
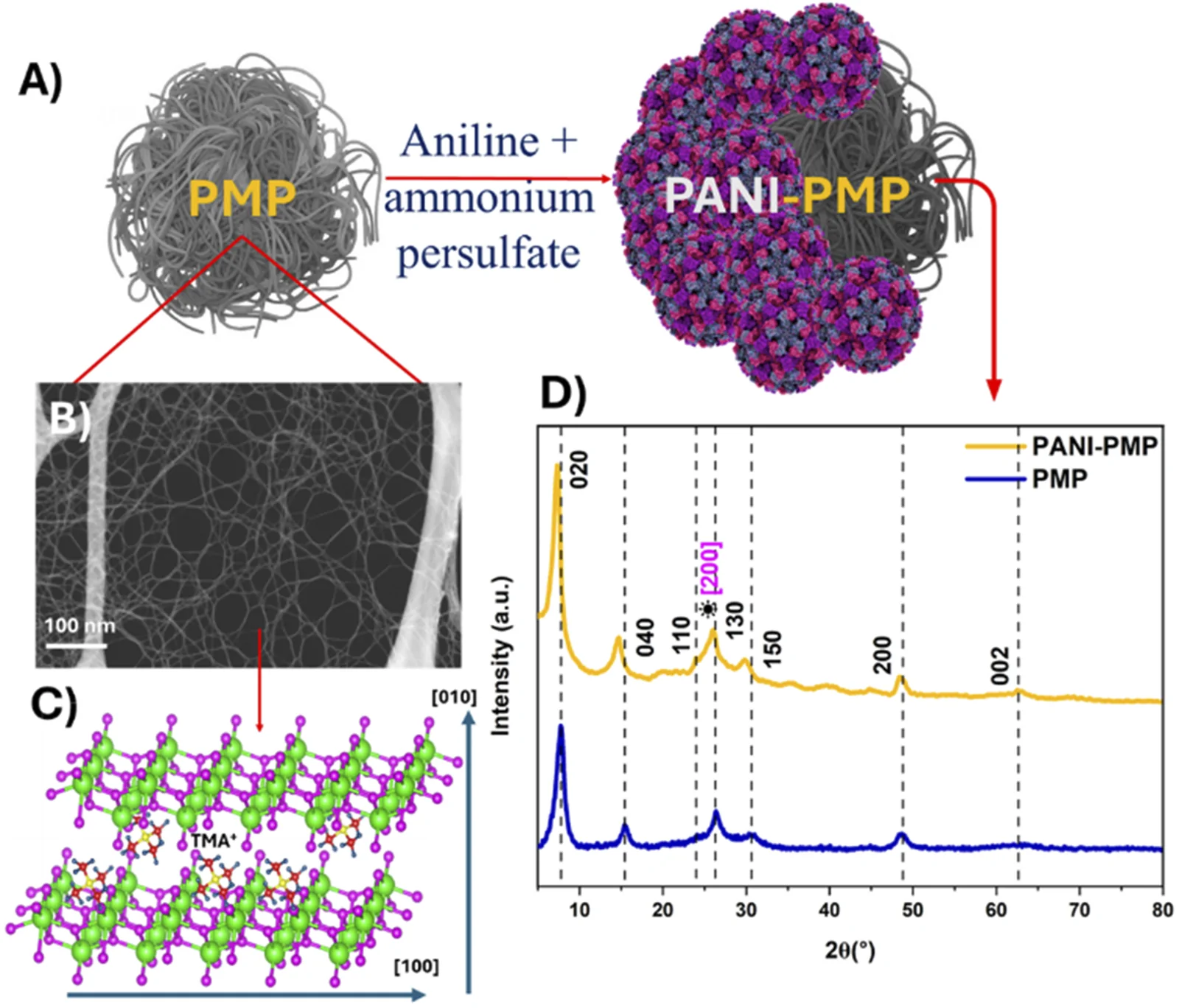
(A) Scheme for converting PMPs into PANI–PMPs through *in situ* polymerization, (B) STEM image of 1DL NFs, (C) schematic of 1DL intercalated with TMA^+^. (D) XRD patterns of the PMP and PANI–PMPs. Labels and curves are color-coordinated.

## Experimental details

### Chemicals

Titanium oxysulfate, TiOSO_4_, tetramethylammonium hydroxide (TMAOH), acetonitrile (ACN) and polytetrafluoroethylene (PTFE) were procured from Sigma-Aldrich and used as received. Polyvinylidene fluoride (PVDF), ammonium persulfate, tetraethylammonium tetrafluoroborate (TEABF_4_), carbon black and *N*-methyl-2-pyrrolidone (NMP) were purchased from Thermo Fisher Scientific. Ethanol (EtOH 200 proof) was obtained from VWR chemicals.

### Synthesis of TiO_2_ PMPs

The TiOSO_4_ precursor powder was mixed with 25 wt% TMAH aqueous solution in a molar ratio of Ti : TMA of 0.4 in a polyethylene bottle. The mixture was heated at 80 °C for 24 h. The resultant mixture was washed with EtOH several times to remove excess TMA (until the pH was ≈7 in EtOH) and other reaction products and air dried. This procedure resulted in PMPs in the form of a free-flowing powder.

### Preparation of PANI–PMP composites

The PMPs (1 g) were mixed with aniline (1 ml) in 1 M HCl solution. The solution was kept in an ice bath and stirred at 500 rpm. After a few minutes, ammonium persulfate (2.28 g) was added slowly to the reaction 1 M HCl mixture. At this junction, the solution changed its color from milky white to dark green, indicating the start of the polymerization (as shown in Fig. S1). The reaction was allowed to proceed for 12 h. Lastly, the resultant suspension was filtered and washed several times to remove unreacted aniline and ammonium persulfate. The resulting powder was air-dried and used for further characterization. For the SC electrodes, we utilized a higher loading of PANI, specifically a PANI–PMP composite with a weight ratio of 2 : 1 respectively.

### Characterization of materials

The composite morphologies were imaged using a field emission scanning electron microscope, SEM (FESEM, Zeiss; Model: Sigma 300, Germany) at varying magnifications. Qualitative elemental analysis was carried out using an energy dispersive X-ray spectroscope (EDS) (Oxford Instruments X-Max, 80 mm^2^ equipped with a silicon drift detector) attached to the SEM. The structure and crystallinity were analyzed using powder X-ray diffraction, XRD (Panalytical; model: X'Pert, Netherlands). The scans were conducted in the 2*θ* range from 5 to 80°, with a step size of 0.02° and a scan rate of 0.5 s per step. The radiation used was CuK_α_ (40 kV, 40 mA).

The N_2_ adsorption–desorption (surface area and porosimeter measurements) was evaluated *via* a particle size analyzer (Micromeritics, Model: ASAP2020, USA).

X-ray photoelectron spectroscopy, XPS, measurements were performed on cold-pressed samples using a surface analysis system (Kratos AXIS Ultra^DLD^, Manchester, U.K.) using monochromatic Al-Kα (1486.6 eV) radiation. The X-ray beam irradiated the surface of the sample at an angle of 45°, with respect to the surface and provided an X-ray spot size of 300 × 800 µm. Charge neutralization was performed using a co-axial, low energy (∼0.1 eV) electron flood source to avoid shifts in the recorded binding energy, BE. The survey spectra were recorded using 160 eV pass energy with 0.1 eV step size. High-resolution XPS spectra were recorded for Ti 2p, C 1s, O 1s and N 1s. The analyzer pass energy used for all the regions was 20 eV with a step size of 0.1 eV. The BE scale of all XPS spectra was referenced to the C/C–H peak at 284.8 eV.

Transmission Fourier transform infrared (FTIR) spectroscopy was performed using a Bruker Vertex 70 spectrometer (Ettlingen, Germany), with a DLaTGS detector, while the system was continuously purged with N_2_. Spectra were acquired at 2 cm^−1^ resolution, adding 64 scans between 5000 and 500 cm^−1^. To prepare the samples for measurements, 300 mg of KBr powder preheated at 100 °C for 1 h (to remove moisture) was ground in a mortar together with 1 mg of PMP and PANI–PMP samples. A pellet was formed under 9 tons of force in a 10 mm die. Samples were then kept for 2 h at 100 °C prior to spectroscopy. The chamber was purged with N_2_ for 15 minutes prior to each reading to ensure removal of ambient moisture and CO_2_. Ambient air was initially measured, followed by a nitrogen-purged sample measurement.

Samples for transmission electron microscope (TEM) samples were prepared using a dual-beam focused ion beam (FIB) system (Thermo Scientific Helios 5 UC DualBeam). A protective Pt layer (∼1 µm thick) was first deposited on the region of interest using electron beam-induced deposition, followed by ion beam-induced deposition, to minimize ion beam damage. Trenches were milled on both sides of the region using a Ga^+^ ion beam at 30 kV, and the lamella was thinned down to approximately 100 nm. The lamella was then lifted out and attached to a Cu half-grid using Pt deposition. Final thinning and cleaning were carried out at 5–2 kV to reduce surface damage.

STEM-EDX characterization of samples was performed using a Linköping double-corrected and monochromated FEI Titan^3^ 60–300 STEM equipped with a SuperX EDX system and operated at 300 kV. This system was used for structural and elemental characterization of the composites.

Raman spectroscopy was performed using a 532 nm DPSS laser (λ-beam, RGB Photonics) with an output power of 5 mW. A spectrograph (Andor Kymera 328i) with a 600 mm^−1^ diffraction grating (blazed at 500 nm) was used for analysis. The system was coupled with an inverted microscope (Nikon Ti-E) with a 60× air objective (Nikon, NA = 0.7). The signal was collected using an EMCCD camera (Andor Newton DU970P-BVF).

### Electrode preparation and electrochemical measurements

The active material, PANI–PMP (2 : 1, respectively) (80%), was mixed with PVDF (10%) and acetylene black (10%), and a slurry was prepared with the help of a NMP solution. Acetylene black is used to form a percolation network that facilitates the flow of electrons, reducing contact resistance between the particles of the active material. The slurry was then drop-cast on a carbon cloth (of a diameter of 10 mm), which served as the supporting electrode substrate. The mass of the active material on the carbon cloth was ≈5.0 mg cm^−2^. All configurations used a glassy carbon (except in coil cells) electrode (CHI instruments, China) as a current collector and a glass microfiber membrane as the separator (Whatman, General Electrics, USA). An AC disc (with 10 wt% PTFE binder) was used as the counter electrode. For three-electrode measurements, the specific capacity is normalized to the mass of the active PANI–PMP material in the working electrode (80% of the total weight). For the symmetric two-electrode cell, the reported device-level quantities are normalized to the combined active-material mass of both electrodes. For the asymmetric PANI–PMP//AC device, the combined active masses of the positive and negative electrodes are used. The binder and conductive additive masses are not included in the reported normalized values. An Ag wire was used as a quasi-reference electrode. The fabricated electrode materials were subjected to cyclic voltammetry (CV), galvanostatic charge–discharge (GCD), and electrochemical impedance spectroscopy (EIS). These experiments were carried out in three-electrode systems immersed in 1 M TEABF_4_ in ACN solution in a Swagelok cell. In the three-electrode system, the mass of the counter electrode is balanced with that of the working electrode. Subsequently, the SC cell characteristics were evaluated in a two-electrode arrangement in a coin cell with 10 µl of electrolyte solution. The execution of all electrochemical assessments was recorded with a potentiostat (VSP, Biologic, France). The appropriate equations, S1–S8, found in the SI, sections were used to calculate the electrodes' capacitances, specific energies, and power.

## Results and discussion

### Material structure

The XRD patterns of the PANI–PMP composite (1 : 1 wt ratio) and pristine PMP (Fig. 1D) provide insight into their crystallinity. 1DL NFs grow in the [100] direction and stack along the [010] direction (Fig. 1C).^9,11^ The low-angle peak (2*θ* = 7.7°) designated as (020) corresponds to a *d*-spacing of 11.4 Å, representing the center-to-center distance between two adjacent 1DLs. Additionally, the peak observed at a 2*θ* value of approximately 48° is indexed as (200), while the peak at 2*θ* of about 62° is identified as a (002) peak. The lattice parameters derived from these peaks are *a* = 3.78 Å and *c* = 2.96 Å, which are in excellent agreement with those reported for 2D lepidocrocite, in general, and 1DLs in particular. The diffraction pattern of the composite (top pattern in Fig. 1D) consists of a broad peak centered at 26° attributed to reflections from the PANI (200) plane^45^ and (130) PMP planes.^11^ Additionally, the characteristic peaks of PMPs shifted slightly toward lower 2*θ* angles as a result of lattice expansion due to the introduction of PANI.

The formation of PANI during polymerization is further confirmed by FTIR spectroscopy. Fig. S2 shows the transmittance peaks of PMPs from 400 to 720 cm^−1^, attributed to Ti–O–Ti bonds.^46,47^ The composite with additional peaks at 3442, 1565, 1468, 1295, and 1107 cm^−1^ can be ascribed to N–H, C=N, C=C, C–N, and C–H bonds, respectively, confirming the contributions from PANI.^47,48^ All the stretching vibration bands of PANI match well with the available literature.^47^ However, there are obvious shifts in the vibration bands due to the synergistic effects between the NFs and PANI matrix. The peaks for N–H, C–N, C=N, and C–H shift to a higher wavenumber, indicating that the bonds become stronger after composite formation, while the C=C peak shifts to a lower wavenumber, suggesting that this bond becomes weaker. These results further affirm the presence of PANI and PMPs in the composite.

The BET (Brunauer–Emmett–Teller) model was employed to calculate the specific surface areas, SSAs, and pore size distributions (Fig. S3) using the N_2_ adsorption–desorption results at 77 K. The SSAs and corresponding pore volumes in pristine PMPs and PANI–PMP composites measured by the BET method are summarized in Table S1. The SSAs of the PMPs and PANI–PMP composite (1 : 1 wt ratio) are calculated to be 98 m^2^ g^−1^ and 23 m^2^ g^−1^ with pore volumes of 0.19 and 0.12 cm^3^ g^−1^, respectively. The theoretical SSA values of the 1DL NFs are in the order of ∼1500 m^2^ g^−1^, assuming that every filament surface is exposed and accessible.^49^ However, the experimental BET value is derived from N_2_ adsorption on self-assembled PMPs, where nanofilaments are tightly packed and partly aggregated. During self-assembly, a large part of the filament surface becomes covered at contact points or trapped within very narrow interfilament spaces that N_2_ molecules cannot reach effectively. Additionally, closed pores, restricted diffusion, incomplete degassing, and the limitations of the BET model can further decrease the measured accessible surface area. The PMP and PANI–PMP composite isotherms exhibit a combination of Type II and III isotherms (Fig. S3) with an H3-type hysteresis loop according to the IUPAC classification.^50^ Fig. S3A shows the rapid uptake at lower relative pressures (*P*/*P*_0_), suggesting complete monolayer coverage of the gas molecule within the pores, and small hysteresis loops at the higher relative *P*/*P*_0_ regions. The latter is most probably due to capillary condensation in the PMP mesopores. The presence of an abundance of mesopores is further confirmed *via* the pore size distributions, shown in the inset in Fig. S3A (pore size 1–10 nm). However, the composite exhibits a decrease in mesopores and an increase in macropores (>10 nm) presumably due to the polymer wrapping around PMP (inset Fig. S3B and 1B).

Typical SEM micrographs of the PANI–PMP composite (1 : 1 wt ratio), at various magnifications, are shown in Fig. 2. The composite displays a similar morphology to pristine PMPs, *i.e.*, a non-agglomerated structure (Fig. 2A), which is why the composites behave as free-flowing powders. The composite retains an almost spherical morphology, with diameters spanning a wide particle size range of ≈1–50 µm. The morphologies appear to consist of polymer-wrapped PMPs. Fig. 2B, C and E display a highly interconnected porous structure that is reminiscent of a cauliflower surface. It follows that synthesis of these composites through *in situ* polymerization provides a unique architecture for ion exchange at the PANI/PMP interface during redox reactions. Fig. 2D and F showcase the self-assembly of the NFs and porous polymer, creating the PANI/PMP intersection.

**Fig. 2:**
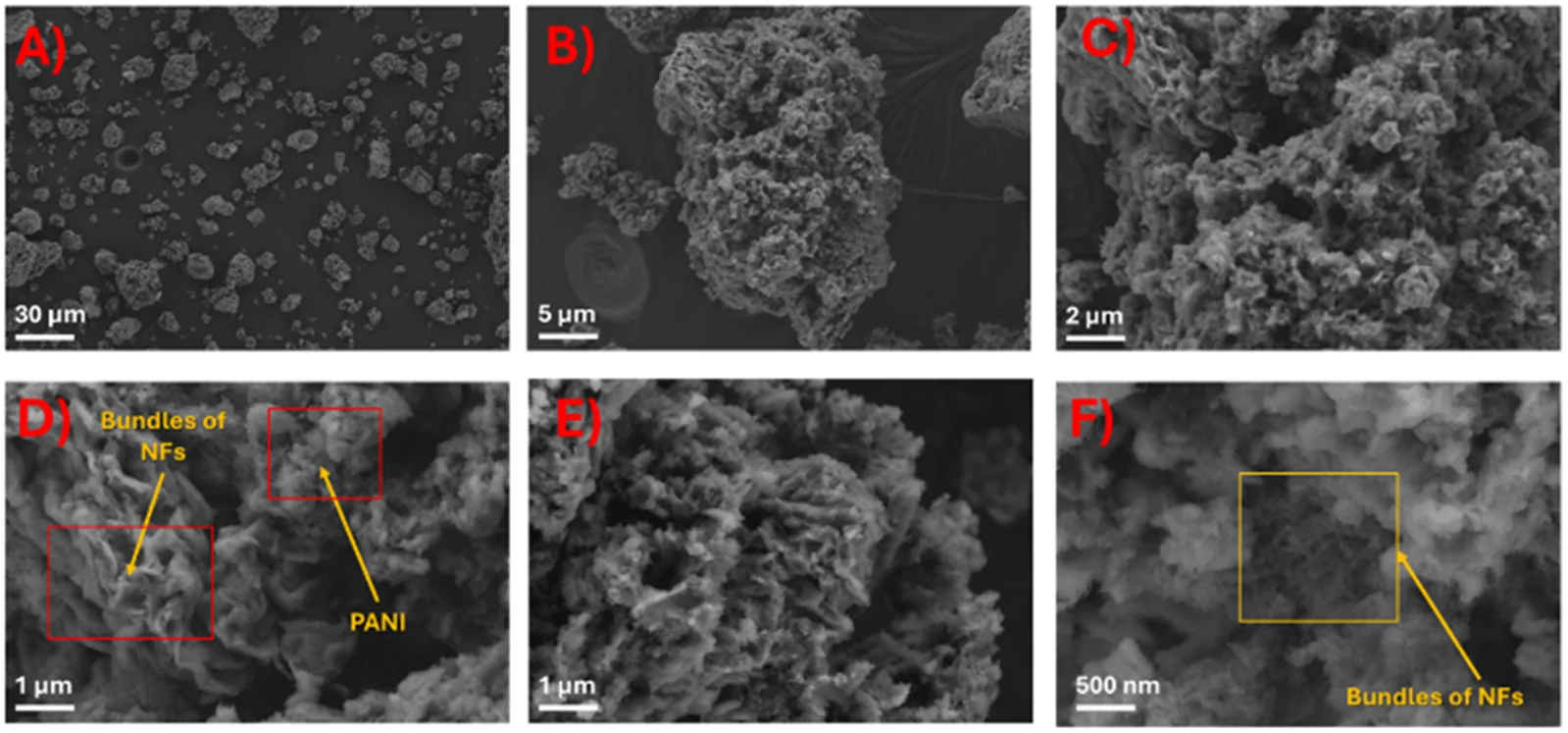
SEM micrograph of the PANI–PMP composite at different magnifications (A–F).

Composite SEM micrographs with higher polymer loadings (in 2 : 1 wt ratio) are shown in Fig. S4 at various magnifications. In this case, the morphology of the porous cauliflower is more distinct. However, bundles of NFs at the PANI edges are not visible due to the lower quantity of the former.

To explore the elemental distribution of the composite, SEM-EDX elemental mapping was performed. Fig. S5 displays the SEM image along with the distribution maps for Ti, O, N and C for both compositions. These results confirm the presence of Ti and O from PMPs as well as C and N from the PANI at the various concentrations in the composite.

Fig. 3A provides a cross-sectional STEM overview image of a microcluster, where 1DLs (denoted as 1) are embedded within the PANI matrix. The porous nature of the structure is clear, with 1DLs (marked as 2) wrapped within the PANI. At higher magnification (Fig. 3B and C), the brighter regions (region 1 in Fig. 3A) correspond to the 1DLs, which are further confirmed from the EDS maps (Fig. 3D). Fig. 3D, E, F and G show the elemental distribution of Ti, O, N, and C, respectively, allowing for a clear differentiation between the bundles of 1DLs and the polymer matrix. The contrast between bright and dark areas in the images originates from differences in atomic number, with Ti being heavier than the polymer matrix. It is evident that the NFs are embedded within the PANI matrix.

**Fig. 3:**
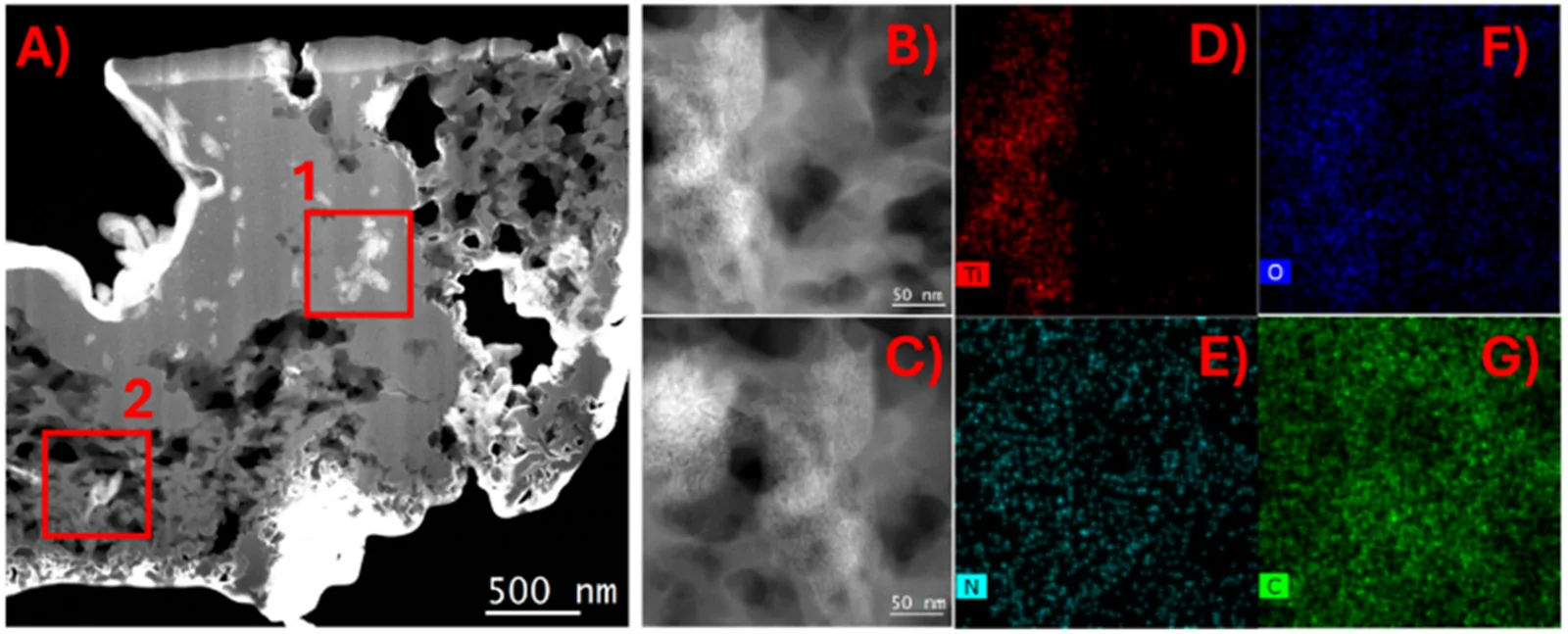
HAADF-STEM images of polyaniline-lepidocrocite titania at varying magnifications (A–C) and STEM-EDS maps of (B) highlighting Ti, O, N, and C distribution (D–G).

The XPS results of the PMP and PANI–PMP composite (1 : 1 wt ratio) cold-pressed samples are shown in Fig. 4 and summarized in Tables 2 and S2. The spectra of the Ti 2p region for both samples can be fitted with one set of peaks assigned to Ti^4+^ at binding energies (BEs) of 459.3 eV for PMP and 459.4 eV for the composite sample. The difference in BE is within the statistical noise, which leads us to conclude that there is no chemical interaction between the PMPs and PANI. The XPS spectra of the O 1s region for PMP and the PANI–PMP composite sample can be fitted into three peaks corresponding to TiO_2_, O belonging to carboxylic groups, which come from the exposure of the sample to the ambient and/or during synthesis, and a third peak is assigned to adsorbed water. The N 1s region PANI–PMP composite sample can be fitted into two peaks corresponding to polyaniline and the ammonium group.

**Fig. 4:**
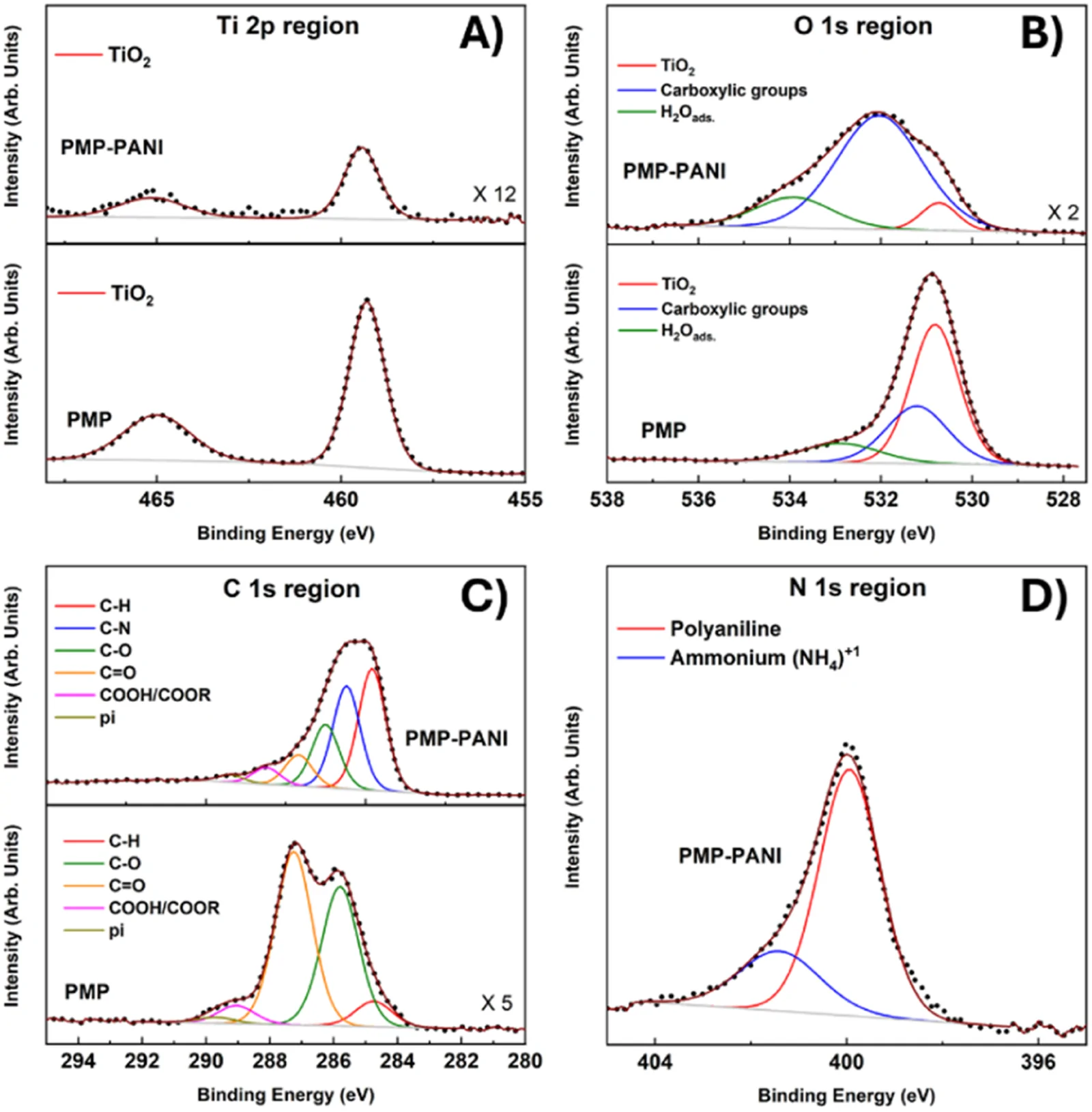
XPS spectra of (A) Ti 2p, (B) O 1s, (C) C 1s, and (D) N 1s regions for PMP and the PANI–PMP composite.

**Table 2:** Summary of global atomic percentages obtained from the XPS survey spectra of PMP and PANI–PMP cold-pressed discs

	Ti	C	O	N	Cl
PMP	16.2 ± 0.2	37.6 ± 0.5	42.1 ± 0.4	4.1 ± 0.4	<0.1
PANI–PMP	1.3 ± 0.3	76.7 ± 0.6	11.2 ± 0.3	9.6 ± 0.5	1.2 ± 0.1

### Electrochemical performance

The electrochemical properties of our PANI–PMP composite were evaluated using CV in 1 M TEABF_4_ in acetonitrile solution in a 3-electrode arrangement. Preliminary results found that PANI–PMP in a 2 : 1 weight ratio was better than its 1 : 1 counterpart, which is why the focus in the following discussion will be on the 2 : 1 composition. The CV response, shown in Fig. S6A, was obtained at scan rates ranging from 2 to 100 mV s^−1^. The results indicate pseudocapacitive and diffusion-controlled faradaic behavior due to the PANI, along with a pair of redox peaks at 0.17 and 0.63 V *versus* Ag wire, corresponding to the reduction and oxidation of Ti^4+^. The operating potential of this electrode is from −1.5 V to 2 V. The highest value of the specific capacity of the composite is calculated to be 364 mA h g^−1^ at a scan rate of 2 mV s^−1^. At that same rate, the swift ionic diffusion creates maximum redox-active sites, leading to higher values of the specific capacitance. Fig. S6B exhibits the GCD curve of the PANI–PMP electrode at current densities varying from 0.5 to 5 A g^−1^. The non-linear voltage plateaus represent dominant faradaic charge storage. The highest capacity of the composite, as calculated from the GCD curves, is 377 mA h g^−1^ at 0.5 A g^−1^. Furthermore, the kinetic parameters of the fabricated electrode were analyzed using EIS measurements. Fig. S6C displays the Nyquist plots measured over the 1 mHz to 1 MHz frequency range. The equivalent series resistance, estimated from the real *Z* intercept, is 3.8 Ω. The charge transfer resistance, *i.e.*, the diameter of the semicircle in the high-frequency region, is around 18 Ω. Furthermore, Fig. S6D shows the Bode plot in the same frequency zone. The crossover frequency of the electrode is observed at 3.2 mHz. These findings illustrate the high potential window, enhanced specific capacity, and faster ion transport of the working electrode.

The PANI–PMP composite was then assembled in a symmetric cell (Fig. 5A), operating at an extensive working voltage of 3.5 V, which is more than double that of typical aqueous electrolytes. The safe working potential was determined from a series of CV curves at 20 mV s^−1^ (Fig. S7A), over an increasing voltage range from 0 to 2 to 3.5 V. The area under the CV curve expands with potential, showing no signs of polarization within the 0–3.5 V potential window. Furthermore, chronoamperometric measurements were performed at progressively increasing cell voltages from 2.0 to 3.5 V (Fig. S7B). At each applied voltage, the current exhibited an initial rapid decay followed by a slower approach toward a quasi-steady value. No continuous increase in current was observed at 3.5 V during the voltage hold, indicating the absence of an obvious runaway decomposition process within the measurement period. However, the residual current increased systematically with applied voltage, indicating increasing faradaic contributions near the upper operating voltage. Fig. 5B plots the CV curves of the composite electrode, demonstrating its roles at both the negative and positive electrodes. A comparison of CVs of pristine PMP and PANI–PMP composite electrodes (Fig. 5C) with capacities of 33 and 436 C g^−1^ at a 2 mV s^−1^ scan rate, respectively, represents a thirteen-fold increase in capacity. The distinct redox peaks for the PANI–PMP composite indicate the faradaic behavior of the electrode. It follows that the inclusion of PMPs in the PANI matrix results in a significantly higher current output (Fig. 5C) and overall capacitance.

**Fig. 5:**
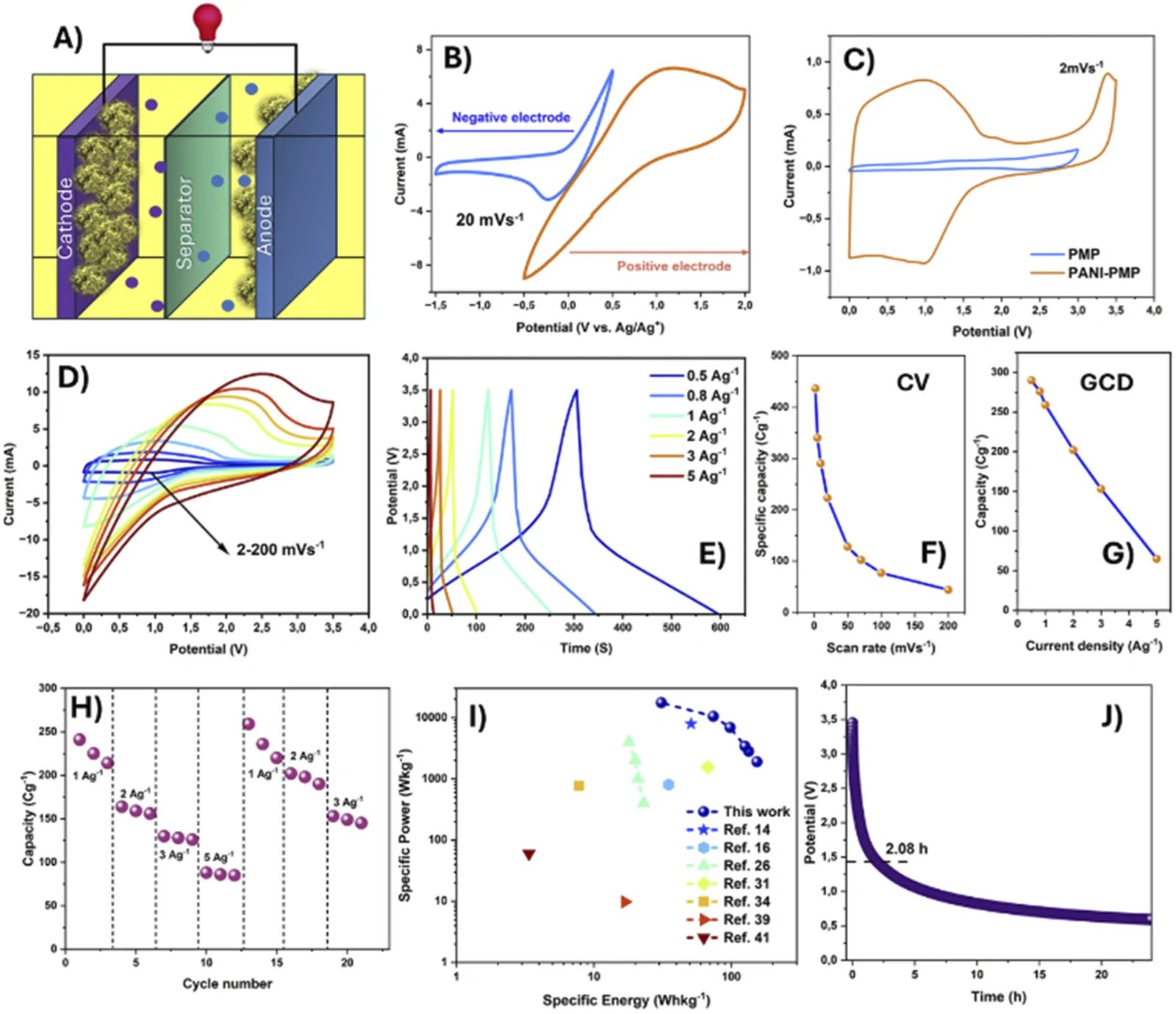
(A) Schematic of the symmetric cell; (B) CV curve of the PANI–PMP composite as the negative and positive electrodes; (C) CV curve of pristine PMP and the PANI–PMP composite at 2 mV s^−1^; (D) CV curves as a function of scan rate; (E) GCD curves at various current densities; Specific capacities at various (F) scan rates and (G) current densities; (H) specific capacities at various current densities during three charge–discharge cycles; (I) Ragone plot of the symmetric SC cell; (J) self-discharge of the cell for 24 h.

Fig. 5D presents the CV response of the symmetric cell at scan rates ranging from 2 to 200 mV s^−1^. As the scan rate increases up to 50 mV s^−1^, both cathodic and anodic peak intensities increase linearly, indicating fast charge transfer kinetics and reversibility. However, at scan rates of 50 mV s^−1^ or higher, the reduction peak intensities decreased due to the faster kinetics. The highest specific capacity was calculated to be 436 C g^−1^ with a specific energy of 212 Wh kg^−1^ at 2 mV s^−1^. Furthermore, the GCD measurement was carried out at varying current densities from 0.5 to 5 A g^−1^, as shown in Fig. 5E. The GCD response reveals a captivating non-triangular shape, beautifully showcasing the dominant and assertive nature of their pseudocapacitance. The discharge region includes a sharp discharge due to faradaic and internal resistances, followed by a voltage plateau. The symmetry of the GCD results demonstrates reversibility and first-rate coulombic efficiency (Table S3). The device's highest specific capacity is calculated to be 290 C g^−1^ at 0.5 A g^−1^. The highest specific energy, at 0.5 A g^−1^, is evaluated at 154 Wh kg^−1^, while maintaining a specific power of 1.9 kW kg^−1^. The highest power of 17 kW kg^−1^ is calculated at a 5 A g^−1^ current density while maintaining a specific energy of 31 Wh kg^−1^. The values of specific capacity, energy and power at varying scan rates and current densities are tabulated in Tables S3 and S4. For comparison, pristine PANI in the same electrolyte has been reported^51^ to exhibit a specific capacitance of ∼150 F g^−1^ with an electroactivity window of 1.0 V, while a symmetric PANI//PANI device delivered 3.5 Wh kg^−1^ and 1.3 kW kg^−1^. These results highlight the substantial enhancement in electrochemical performance achieved upon hybridization of PANI with PMP.

To understand the charge storage mechanisms, we analyzed the results assuming *i* = *av*^*b*^, where *i* is the peak current, *ν* is the scan rate, and *a* and *b* are fitting constants. The ‘*b*’ parameter is indicative of the type of charge storage mechanism. Fig. S7C shows the plot for the PANI–PMP composite, from which *b* is calculated to be 0.39 and 0.57 for the oxidation and reduction peaks, respectively. The redox peaks at 0.75 and 0.47 V (in Fig. S7D) were chosen for the calculation of the diffusion and capacitive control capacitance distribution. A kinetic analysis was also performed to quantify the capacitive and diffusion contributions to the overall capacitance using *i*(*V*) = *k*_1_*ν* + *k*_2_*ν*^1/2^, where *k*_1_ and *k*_2_ are constants that can be calculated by plotting *i*(*V*)/*ν*^1/2^*versus ν*^1/2^.^52^ Fig. S7E summarizes the capacitive and diffusion-controlled distributions of the total capacitances for the SC cell at 1 mV s^−1^. Therefore, the b-values indicate that the redox-peak kinetics involve a surface-controlled pseudocapacitive and a diffusion-controlled faradaic mechanism. However, the influence of diffusion becomes more significant as scan rates decrease (Fig. S7F).

The effect of scan rates on specific capacitance is shown in Fig. 5F. The capacity drops to ≈48% as the scan rate is increased tenfold (2 to 20 mV s^−1^), presumably due to limited diffusion of ions into the active material at the faster kinetics. Furthermore, it drops to 10% as the scan rate is further increased 100 times (2 to 200 mV s^−1^). The effect of current density on specific capacitance (Fig. 5G) shows a linear response where the capacity drops to 22% as the current density increases 10 times (0.5 to 5 A g^−1^). During high rates of charge and discharge, incomplete ion detachment and embedding most probably diminish the pseudocapacitance contributions and overall capacities. The average specific capacities of the device were recorded at current densities of 1, 2, 3, and 5 A g^−1^, with data collected over three cycles for each density, as shown in Fig. 5H. Not surprisingly, the capacitance decreases as the current density increases. However, when the current density is reduced from 5 A g^−1^ back to 1 A g^−1^ after the 12^th^ cycle, a slight increase in capacitance is observed most likely due to the exposure of fresh electrocatalytic active sites resulting from the cycling process.

The Ragone plot of our SC cell and other values in the literature (see Table 1) is shown in Fig. 5I. It follows that our composite enables the delivery of high energy density without compromising power output. The cell delivers the highest energy density of 154 Wh kg^−1^ while maintaining a specific power of 1.9 kW kg^−1^. The highest specific power of 17 kW kg^−1^ is calculated at 5 A g^−1^ current density while maintaining a specific energy of 31 Wh kg^−1^. The Ragone plot compares the performance of TiO_2_-based composites in symmetric and asymmetric cells (from Table 1), showing that our cell outperforms the others with a significant margin.

The self-discharge (SD) of the SC cell is displayed in Fig. 5J. The open-circuit voltage (OCV) drops from 3.5 V to 0.5 V in 24 h. The voltage drops exponentially to 1.48 V (∼42% of the total voltage) in ≈2 h. The SD of the cell is attributed to several factors,^53^*e.g.*, (a) acetonitrile may slowly decompose at the elevated voltages, which may introduce parasitic reactions that contribute to SD; (b) in porous electrodes, charges often do not distribute uniformly. Charge redistribution over time can cause gradual voltage drops, and (c) residual ion transport and leakage currents. The SD would have to be slowed down extensively to expand the application space. This comment notwithstanding, this work will undoubtedly offer crucial insights that will drive the development of high performance electrochemical devices.

EIS presents a deeper understanding of the electrical properties of the SC cell. Fig. 6A presents the Nyquist plots collected over a frequency range of 1 mHz to 1 MHz. The graph exhibits a semicircle at high frequencies with a diameter reflecting the total charge transfer resistance (*R*_CT_ = 15.2 6 Ω) of both electrode–electrolyte interfaces. Furthermore, the near-vertical straight line in the low-frequency region indicates a surface-controlled pseudocapacitive response. The total equivalent series resistance of the cell is calculated to be 10.5 Ω. This value results from the internal resistances of the electrolytes, electrodes, and current collectors. After 10 000 charge and discharge cycles, *R*_CT_ increased to 66 Ω, and the slope in the low-frequency region also decreased (Fig. S7G), likely due to morphological changes in the composite electrode reducing the electrochemically active sites. The equivalent series circuit is shown in the inset of Fig. 6A. The values of the circuit components are listed in Table S5.

**Fig. 6:**
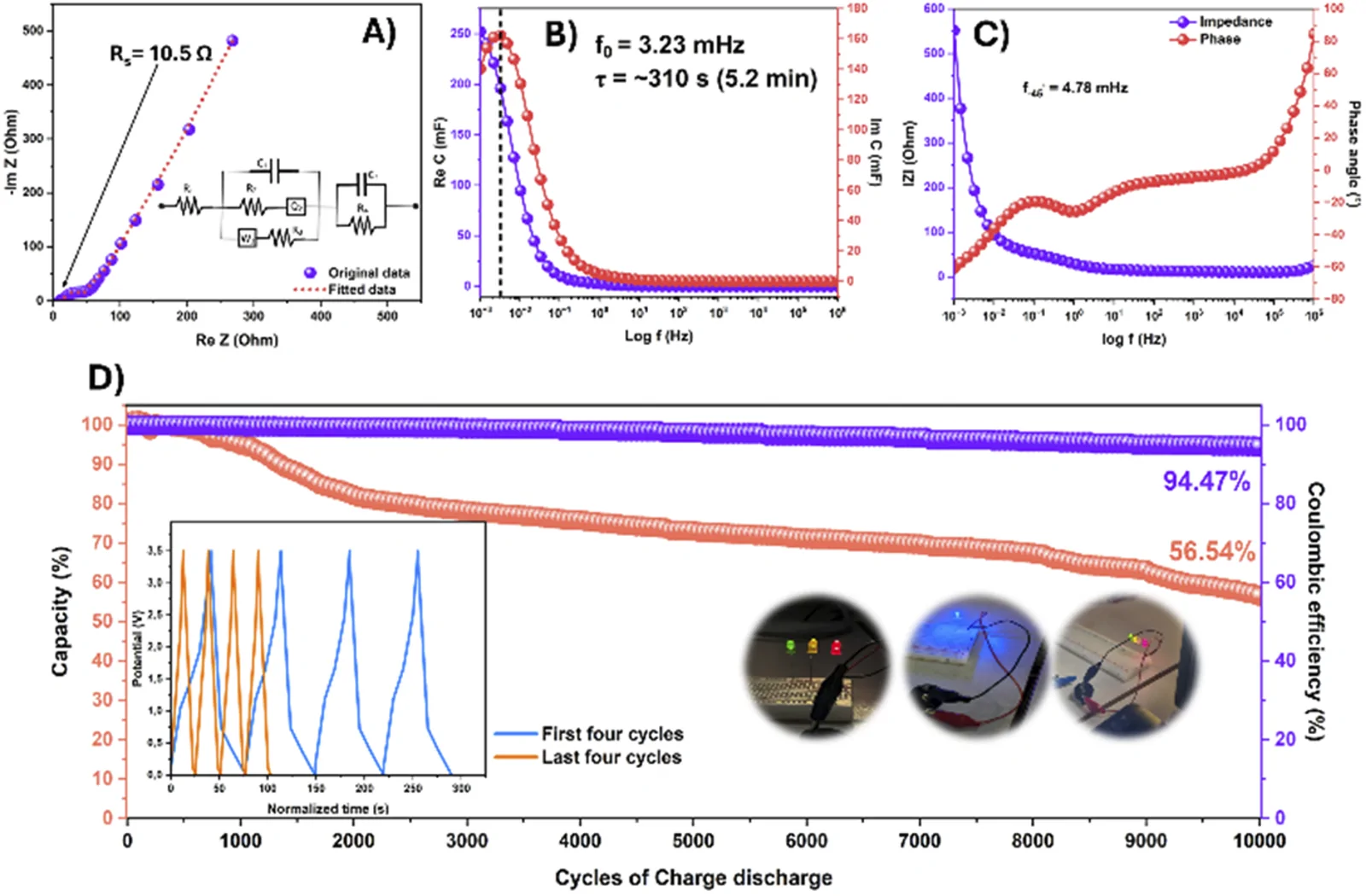
(A) Nyquist plot of the symmetric cell (the equivalent series circuits are plotted as insets); (B) variation of real and imaginary parts of the capacities with frequency; (C) Bode plot; (D) cycle stability and coulombic efficiency of the cell (inset shows the first and last five cycles of the GCD response).

The kinetic response of the cells is characterized by a relaxation time constant (*τ*_0_ = *f*_0_), which is the minimum time required to discharge all the cell energy with 50% efficiency or more. *f*_0_ is the characteristic frequency and is the point at which the imaginary part of the capacitance peaks (Fig. 6B). As shown in Fig. 6B, *τ*_0_ is only ∼310 s (5.2 min), which indicates that the device can store charge under ultrafast switching conditions. Fig. 6C exhibits the Bode plot representing the frequency-dependent variation of impedance and phase. Typically, impedance tends to decrease as the frequency of the signal increases. This relationship highlights how the reactive properties of the circuit change with frequency. Additionally, the phase angle serves as an important indicator, reflecting the relative contributions of surface-controlled pseudocapacitive and resistive behaviors within the cell. The crossover frequency (*f*_−45°_) above which the resistive behavior becomes dominant is 4.78 mHz (Fig. 6C). The cell shows an extended range of electrical and chemical properties that span its application, requiring both rapid energy delivery and substantial energy storage.

The electrochemical cycle stability of the symmetric cell was tested by running 10 000 cycles of charge and discharge at a current density of 5 A g^−1^. As shown in Fig. 6D, the cell retains 73% and 56% of its initial capacitance, respectively, after 5000 and 10 000 cycles. The device holds its coulombic efficiency at 94% after the latter. Such decreases in capacitance during cycling can be attributed to various factors, such as (a) morphological changes in the active material, reducing the electrochemically active sites, and/or (b) degradation of the electrolyte after extensive cycling, which agrees with the EIS results. The increase in charge-transfer resistance from 15.2 Ω to 66 Ω after 10k cycles, together with the decrease in capacitance, indicates progressive deterioration of charge-transfer pathways. The capacity fade arises from a combination of PANI and electrode structural evolution, increased interfacial resistance, and electrolyte/electrode side reactions during extended cycling in an organic electrolyte medium. Nevertheless, the structural integrity of the PANI–PMP composite remains intact, as the response of the last five cycles of charge–discharge corresponds to that of the first five cycles, albeit with different discharge times (as shown in the inset of Fig. 6D).

To reduce self-discharge further and enhance cell stability over multiple charge–discharge cycles, an asymmetric arrangement was designed and investigated using a PANI–PMP composite as the positive electrode (cathode) and activated carbon (AC) as the negative electrode (anode). To achieve maximum efficiency, a balanced charge storage is required between the two electrodes (*q*^+^ = *q*^−^). The optimal mass ratio between the positive and negative electrodes can be obtained using 
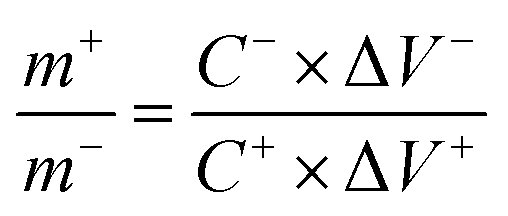
, where (*m*^+,−^), (Δ*V*^+,−^), and (*C*^+,−^) are the mass, operating potential, and specific capacitance of the cathode and anode, respectively. Fig. 7A shows the CV plots of the PANI–PMP and AC in their respective operating potential window. The CV scans of the PANI–PMP//AC cell were carried out at varying scan rates of 2–200 mV s^−1^ in a 3.5 V potential window and are shown in Fig. 7B. The CV profile displays hybrid charge storage with a distorted rectangular shape and broad redox peaks. The cell exhibits the highest capacitance of 94 F g^−1^ at 2 mV s^−1^ with a specific energy of 160 Wh kg^−1^. The capacitance retention as the scan rate changes is illustrated in Fig. S8A, showing that the cell retains 38% of its initial capacitance when the scan rate is increased by a factor of 100 (from 2 to 200).

**Fig. 7:**
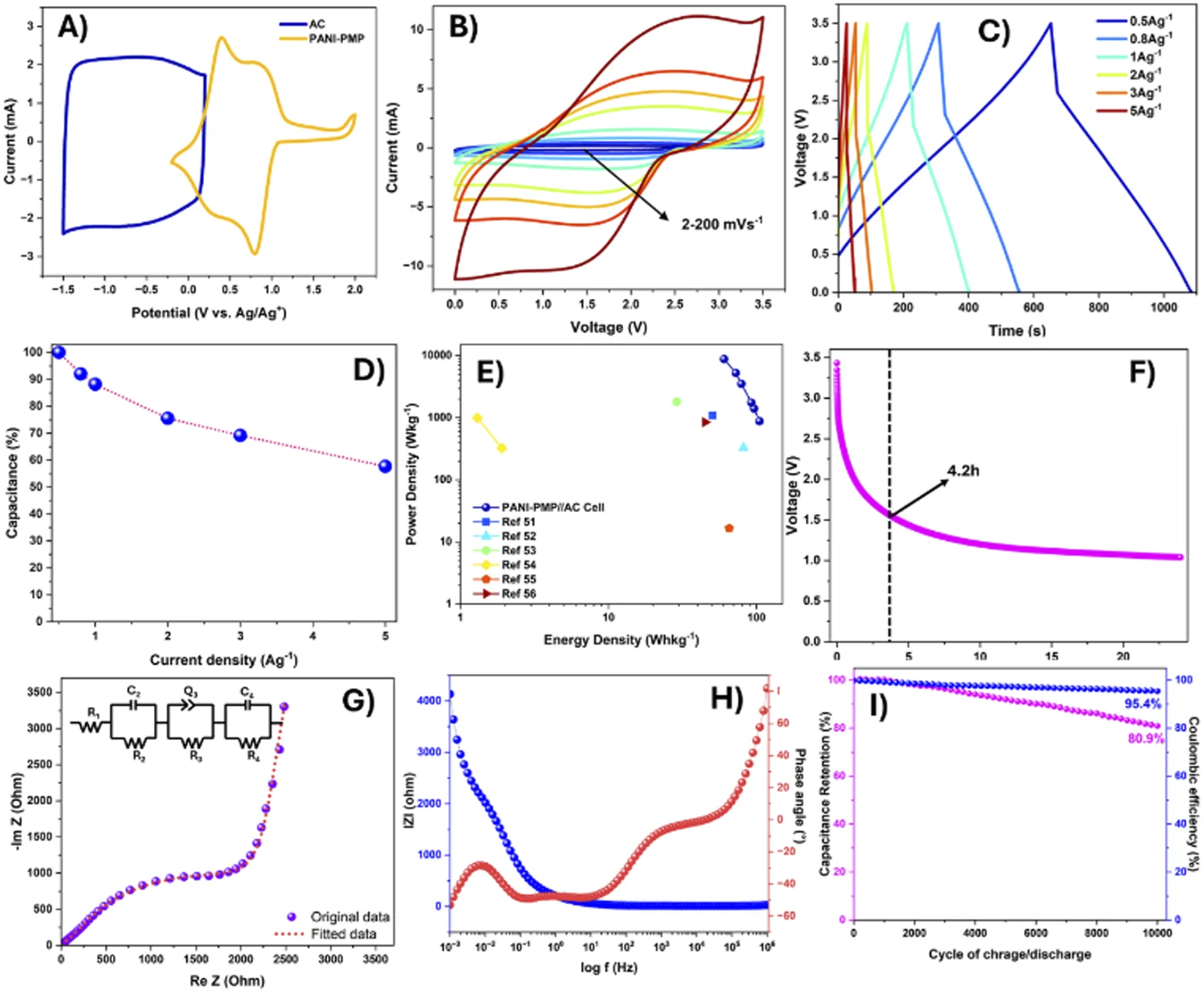
(A) CV curve of AC and the PANI–PMP composite as the negative and positive electrodes, respectively; (B) CV curves as a function of scan rate; (C) GCD curves at various current densities; (D) specific capacities at various current densities; (E) Ragone plot; (F) self-discharge for 24 h; (G) Nyquist plot (the equivalent series circuits are plotted as insets); (H) Bode plot; (I) cycle stability and coulombic efficiency for the PANI–PMP//AC cell.

Fig. 7C presents the GCD profile of the PANI–PMP//AC cell from 0.5 to 5 A g^−1^ current density. The GCD curves are symmetrical and almost triangular. The small *IR* drop occurs due to higher charge transfer and diffusion resistance, which will be discussed later in the impedance analysis. The highest specific capacitance, calculated from the discharge graph (which includes the *IR* drop), is 62 F g^−1^ at a current density of 0.5 A g^−1^ with a specific energy of 105 Wh kg^−1^. The highest specific power is calculated to be 8.7 kW kg^−1^ at 5 A g^−1^ while maintaining a specific energy of 60 Wh kg^−1^. The rate capability of the cell is further evaluated and is shown in Fig. 7D. The cell retains 57% of its initial capacitance when the current density increases from 0.5 A g^−1^ to 5 A g^−1^. Furthermore, a Ragone plot visualizes the cell's performance by showing the trade-offs between energy and power. Fig. 7E compares the energy and power of recently reported AC-based asymmetric SCs with those of the PANI–PMP//AC cell.^54–59^ The highest energy density is calculated to be 105 Wh kg^−1^ with a power density of 0.8 kW kg^−1^ at 0.5 A g^−1^, whereas the highest power density is calculated to be 8.7 kW kg^−1^ while maintaining an energy density of 60 Wh kg^−1^ at 5 A g^−1^. The Ragone plot also expanded to include two-electrode carbon-, metal oxide-, conducting polymer-, and MXene-based SCs in Fig. S8B. The SD of the PANI–PMP//AC cell improved compared with that of the symmetric SC cell, as shown in Fig. 7F. The OCV for PANI–PMP//AC drops from 3.5 to 1.04 V after 24 h. The initial rapid voltage drop occurs within 4.2 h due to rapid relaxation and redistribution of ions at the electrode surface, together with faradaic reactions. After that, a slow voltage decay can be observed due to faradaic or internal leakage. Furthermore, an extended OCV decay measurement was carried out for 120 h, as shown in Fig. S9, and the cell held the potential at 0.43 V. Therefore, the voltage decay is attributed to a combination of charge redistribution/ion diffusion within the porous electrodes, activation-controlled faradaic side reactions at the electrode/electrolyte interface, and residual ohmic leakage.^60–62^

Fig. 7G presents the Nyquist plots collected over a frequency range of 1 mHz to 1 MHz for the PANI–PMP//AC cell. The graph exhibits a steep upward curve in the low-frequency region, indicating diffusion-limited behavior due to mass transport limitations. In the mid-frequency region, a broad semicircle appears due to the charge transfer resistance, indicating that the electrode interface is much more resistive. The linear section in the high-frequency section reflects the solution resistance. The equivalent circuit is illustrated in the inset of Fig. 7G, and the values are tabulated in Table S7. Fig. 7H displays the Bode plot, which represents the frequency-dependent variation of impedance and phase, with a crossover frequency of 50 mHz. The relaxation time, *τ*_0_ = 0.16 ms, is calculated from Fig. S8C, showing ultrafast switching conditions.

Additionally, the stability of the asymmetric cell significantly improved compared to the symmetric one, maintaining nearly 81% of its initial capacitance with a coulombic efficiency of 95% over 10 000 charge–discharge cycles as shown in Fig. 7I. The cell continued running until 100 000 cycles, only to find that the capacitance degradation exceeded 70%. The Nyquist plot (Fig. S8D) reveals a significant increase in resistance after 100 000 cycles, highlighting the expected capacitance loss.

Raman spectroscopy was performed on the as-received carbon cloth and the PANI–PMP structure before and after cycling (Fig. S10A) to understand the change in electrode architecture. In each case, the spectra were averaged from three different regions of the sample (Fig. S10B). The spectra of the pristine carbon cloth exhibit the typical D, G, and 2D modes, corresponding to defect-induced vibrations, in-plane sp^2^ carbon stretching, and a second-order two-phonon scattering process, respectively. Upon addition of PANI–PMP with PVDF, new peaks characteristic of PANI–PMP and PVDF appear, confirming successful coating on the carbon cloth. After cycling, additional bands associated with TEABF_4_ and ACN emerge, along with a C–H stretching feature, indicating the presence of electrolyte residue and minor surface chemical modification. However, no noticeable peak shift or change in the D/G intensity ratio is observed between the spectra before and after cycling, suggesting that no major chemical or microscopic structural alteration occurred. It should be noted that Raman spectroscopy primarily probes local bonding and short-range order; therefore, macroscopic morphological changes, if present, may not be reflected in these spectra.

Furthermore, SEM-EDS analysis assessed the morphological and compositional changes of the PANI–PMP electrode before and after cycling (Fig. S11). The pristine electrode displayed an agglomerated particulate structure with C, N, O, F, and Ti. After cycling, the overall morphology was preserved, but significant changes were noted in surface composition. The fluorine content increased significantly from 2.87 wt% to 15.82 wt%, while titanium decreased from 0.98 wt% to 0.58 wt%. This increase in fluorine correlates with the accumulation of BF_4_^−^-containing electrolyte species during cycling. The C, N, and Ti signals suggest that the PANI–PMP framework was retained, while the drop in Ti levels may be due to masking by electrolyte-derived surface species. The N-signal arises from the TMA^+^ cations. Such surface deposits can hinder ion diffusion and partially block active sites, contributing to increased resistance and gradual capacitance loss.

## Conclusions

In summary, we designed and fabricated a unique composite structure of PANI-wrapped 1DL titania NFs that resulted in a high-energy SC. Structural, morphological, and textural analyses of the composite provide insight into its crystal structure, chemical bonding, atomic distribution, and molar ratios. The concentration of the PANI–PMP composite was optimized, revealing that a 2 : 1 wt% ratio of PANI to PMP exhibits the best electrochemical properties. Benefiting from the synergy of a conducting polymer and redox-active 1D filaments, the composite achieved high capacity (364 mA h g^−1^ at a 2 mV s^−1^). Assembled in a symmetric cell, the device can operate over a wide 3.5 V operating window and delivers a remarkable specific energy of 154 Wh kg^−1^ (the highest among TiO_2_-based electrodes to date), while retaining a high specific power of ∼2 kW kg^−1^ under the same conditions. In the asymmetric arrangement, the PANI–PMP//AC cell shows lower SD, with only a 29.7% open-circuit potential retention after 24 h.

## Author contributions

Sukanya Maity: conceptualization, methodology, investigation, formal analysis, data curation, visualization, writing – original draft. Joseph Halim: data curation, formal analysis. Ali Saffar Shamshirgar: data curation, formal analysis. Risha Achaiah Iythichanda: data curation, formal analysis. Per O. Å. Persson: formal analysis, writing – review and editing. Michel W. Barsoum: supervision, writing – review and editing. Johanna Rosen: supervision, funding acquisition, writing – review and editing.

## Conflicts of interest

There are no conflicts to declare.

## Supplementary Material

TA-OLF-D6TA05686E-s001

## Data Availability

The data supporting the findings of this study are available within the article and its supplementary information (SI). Additional data are available from the corresponding author upon reasonable request. Supplementary information is available. See DOI: https://doi.org/10.1039/d6ta05686e.
