# Supplementary material for: Polyaniline-hybridized quantum-confined 1D lepidocrocite titania for ultra-high-energy supercapacitors
Source: J Mater Chem A Mater. 2026 Nov 9. Online ahead of print. doi: 10.1039/d6ta05686e (PMC13746133; doi:10.1039/d6ta05686e)
Supplement: Supplementary file 1 [file TA-OLF-D6TA05686E-s001.pdf]

Supplementary Information (SI) for Journal of Materials Chemistry A.
This journal is © The Royal Society of Chemistry

ARTICLE

Polyaniline-Hybridized Quantum-Confined 1D Lepidocrocite Titania for Ultra-High-Energy Supercapacitors

Received 00th January 20xx,
Accepted 00th January 20xx

DOI: 10.1039/x0xx00000x

Sukanya Maity[a]†, Joseph Halim[a], Ali Saffar Shamshirgar[a], Risha Achaiah Iythichanda[a], Per O.Å. Persson[a], Michel W. Barsoum[b]†, Johanna Rosen[a]†

Supporting Information

List of Figures:

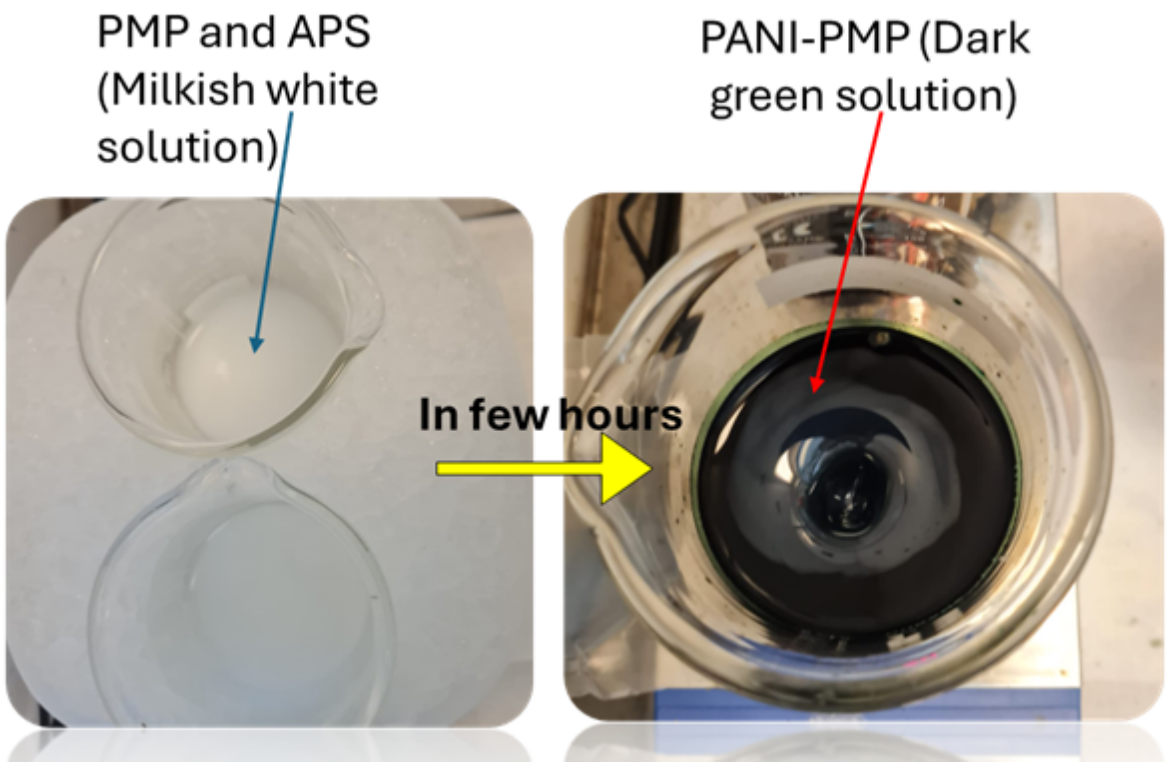

Fig. S1 In-situ polymerization of PMPs to PANI-PMPs

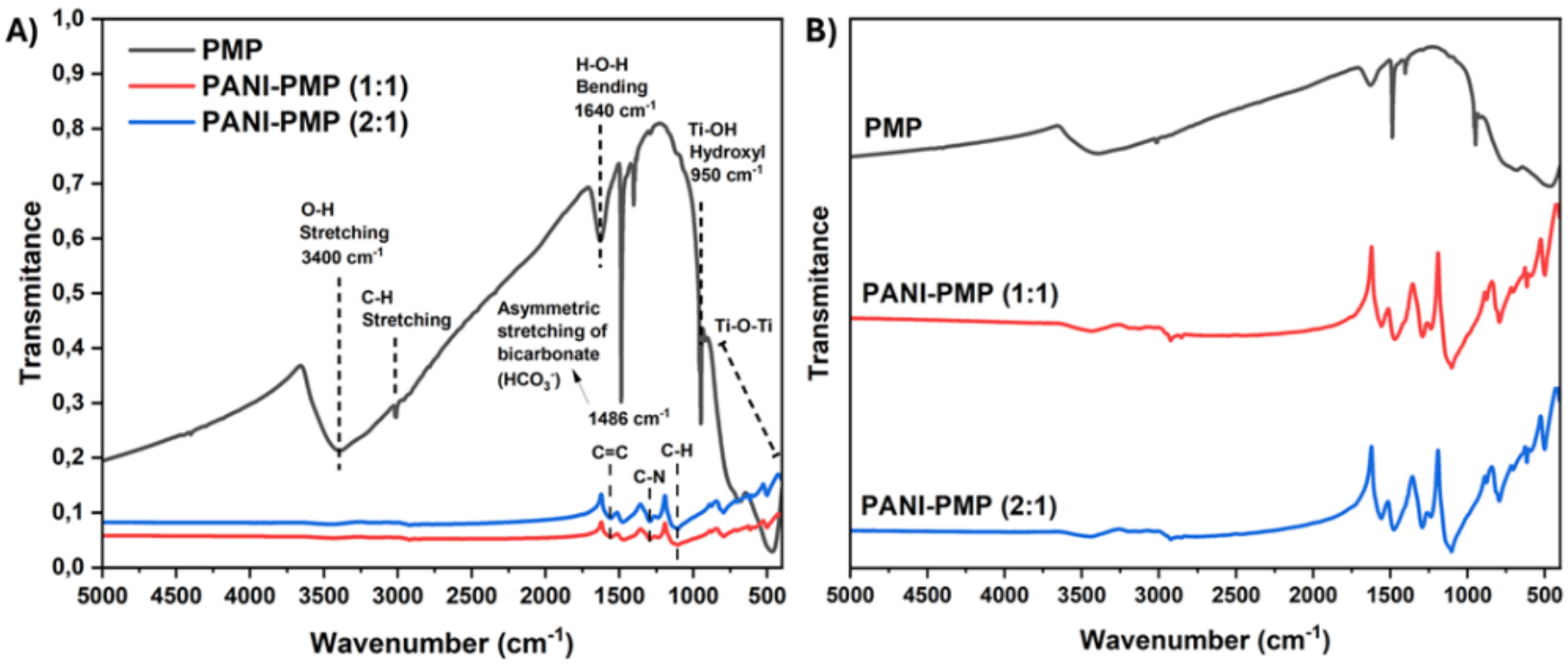

Fig. S2 A) FTIR of the PMP and PANI-PMP composites B) Spectra are normalized from 0-1 for comparison.

[a] Department of Physics, Chemistry and Biology (IFM), Linköping University, 58183 Linköping, Sweden
[b] Department of Materials Science and Engineering, Drexel University, 19104 Pennsylvania, Philadelphia, USA
† Corresponding Author, sukanya.maity@liu.se, barsoumw@drexel.edu, johanna.rosen@liu.se

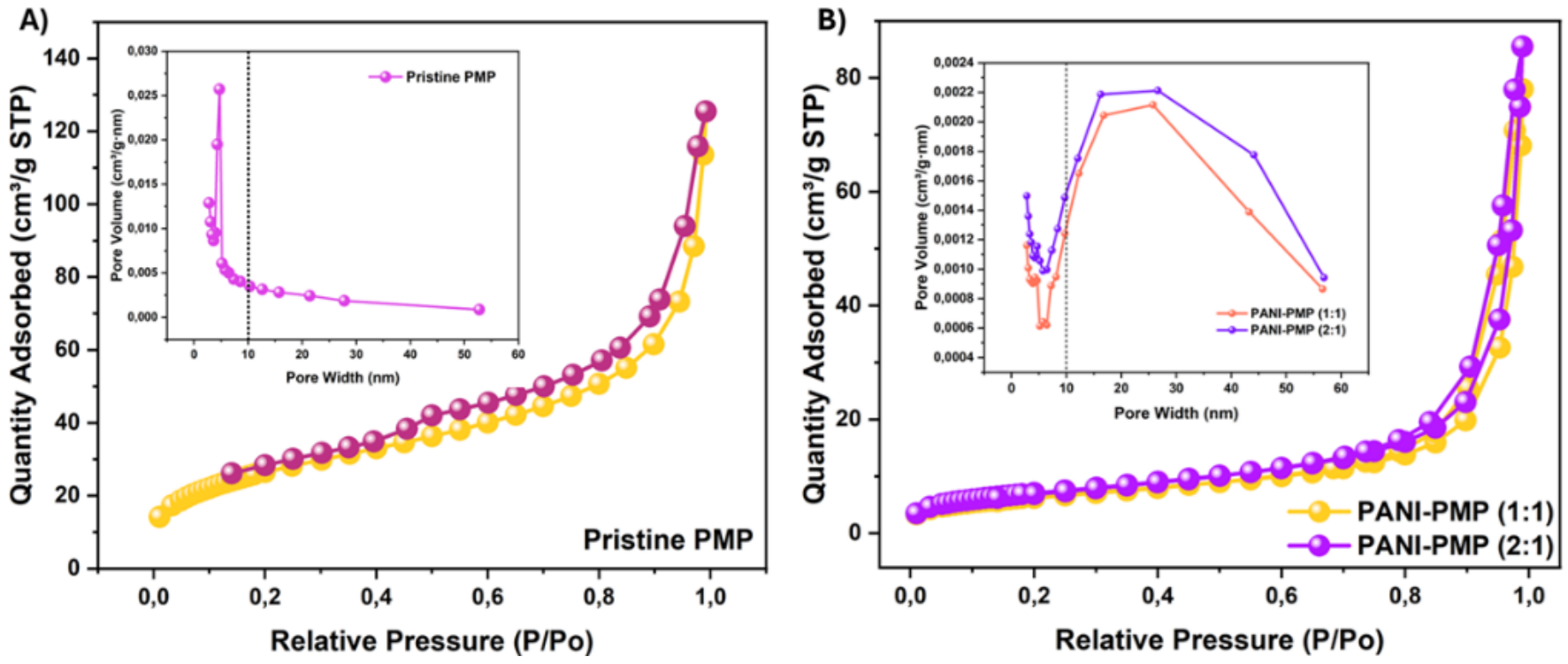

Fig. S3 BET isotherm (inset: pore distribution) of the A) PMP and B) PANI-PMP composites.

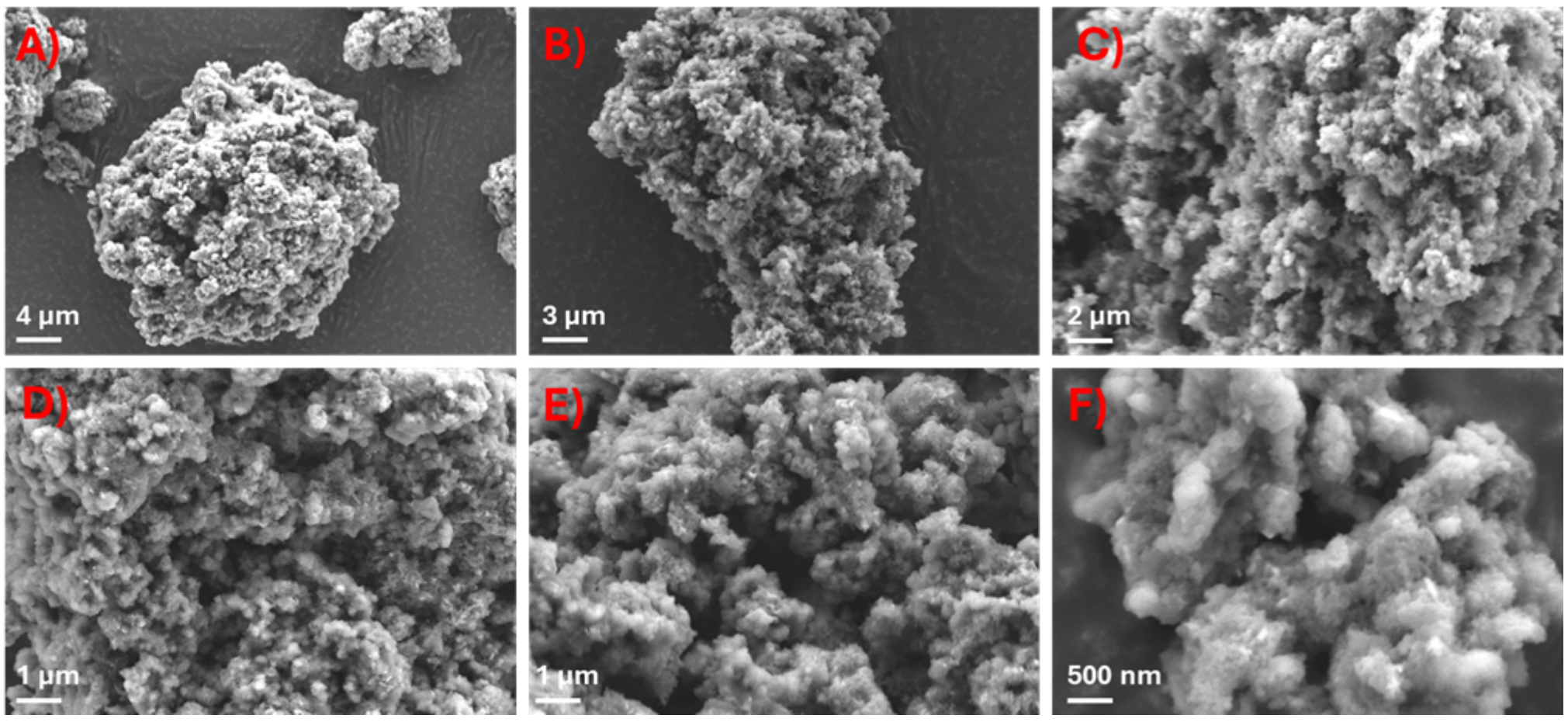

Fig. S4 SEM micrograph of the PANI-PMP composite (2:1)

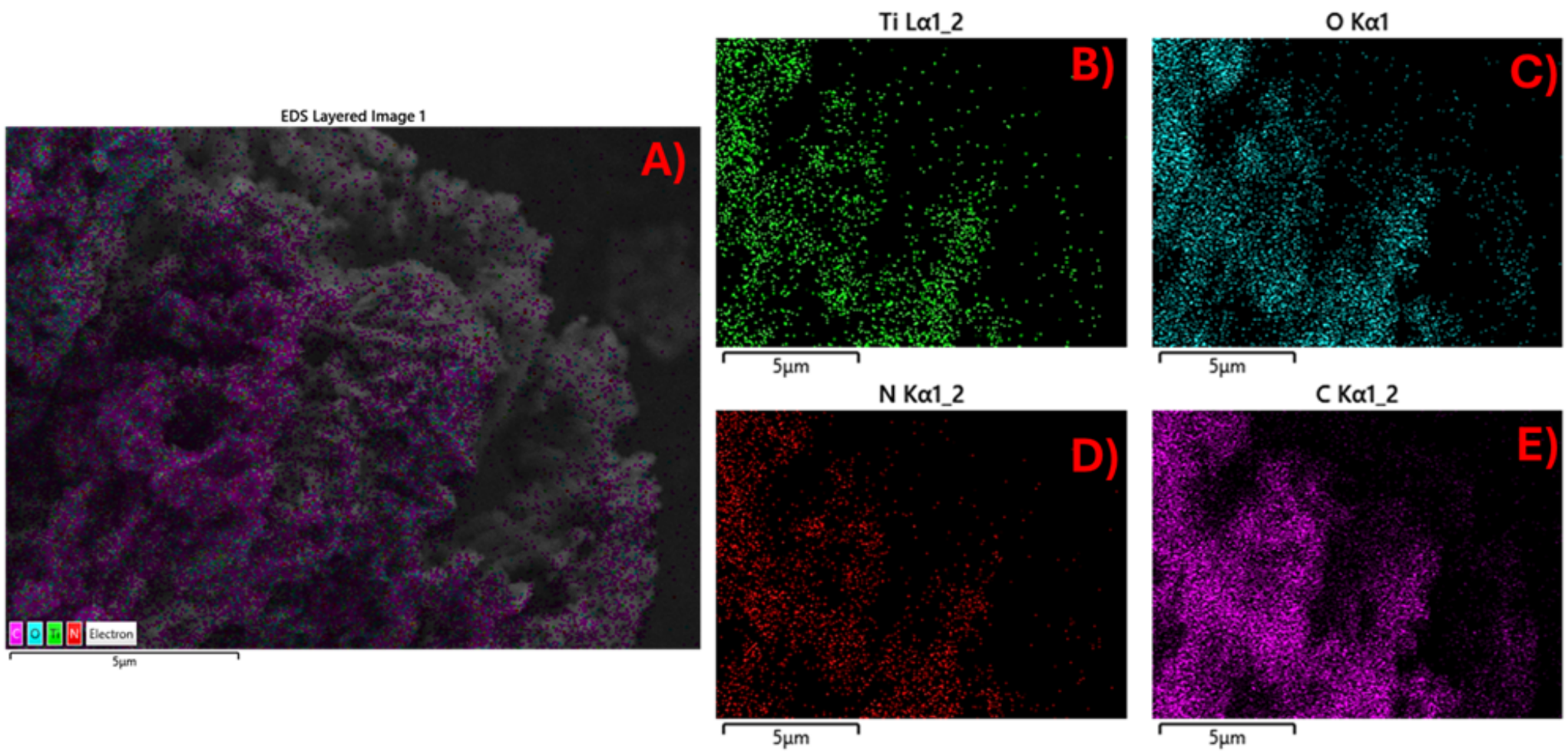

 This journal is © The Royal Society of Chemistry 20xx

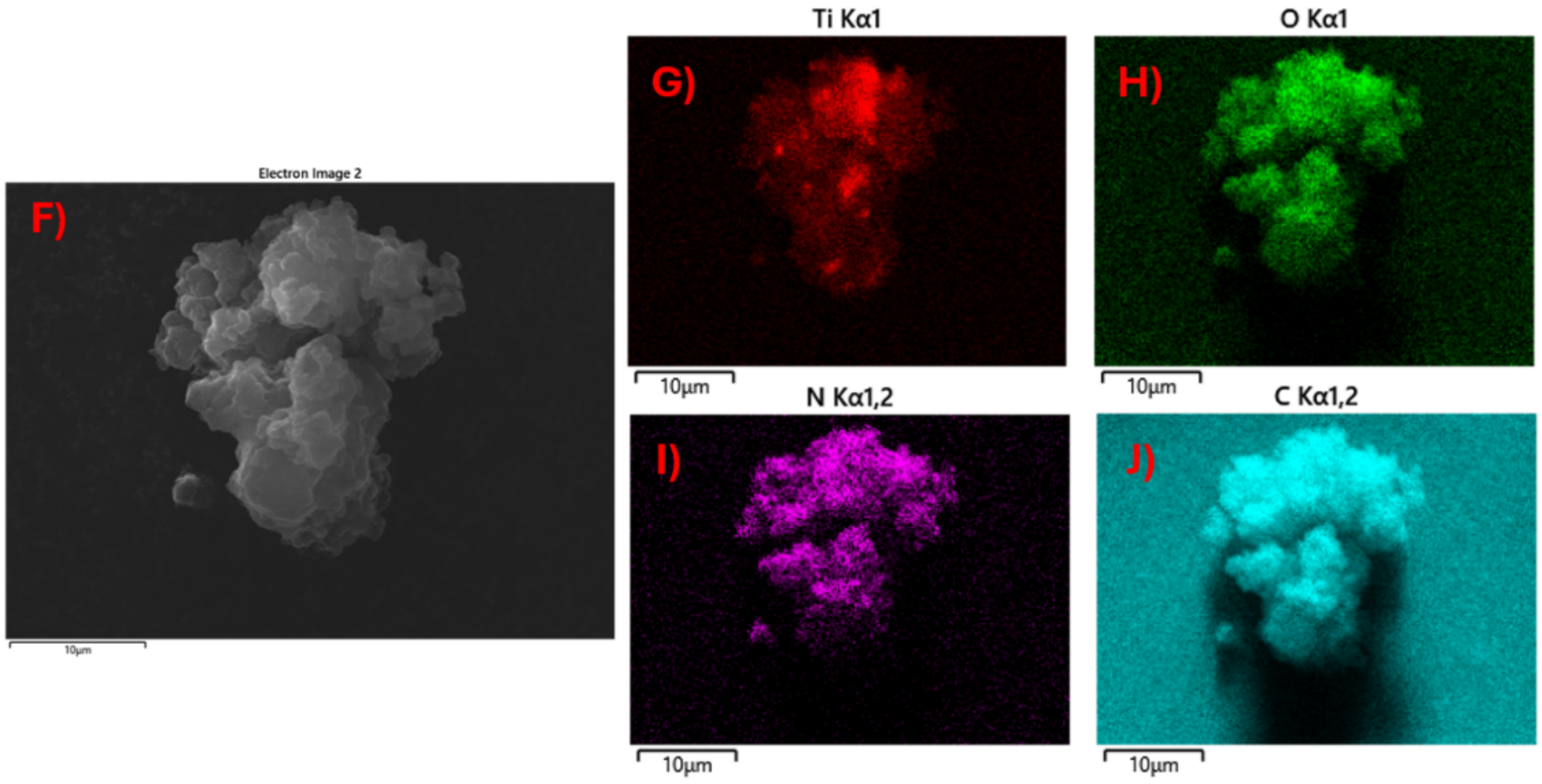

Fig. S5 Elemental mapping of the PANI-PMP composite for 1:1 (A-E) and 2:1 (F-J)

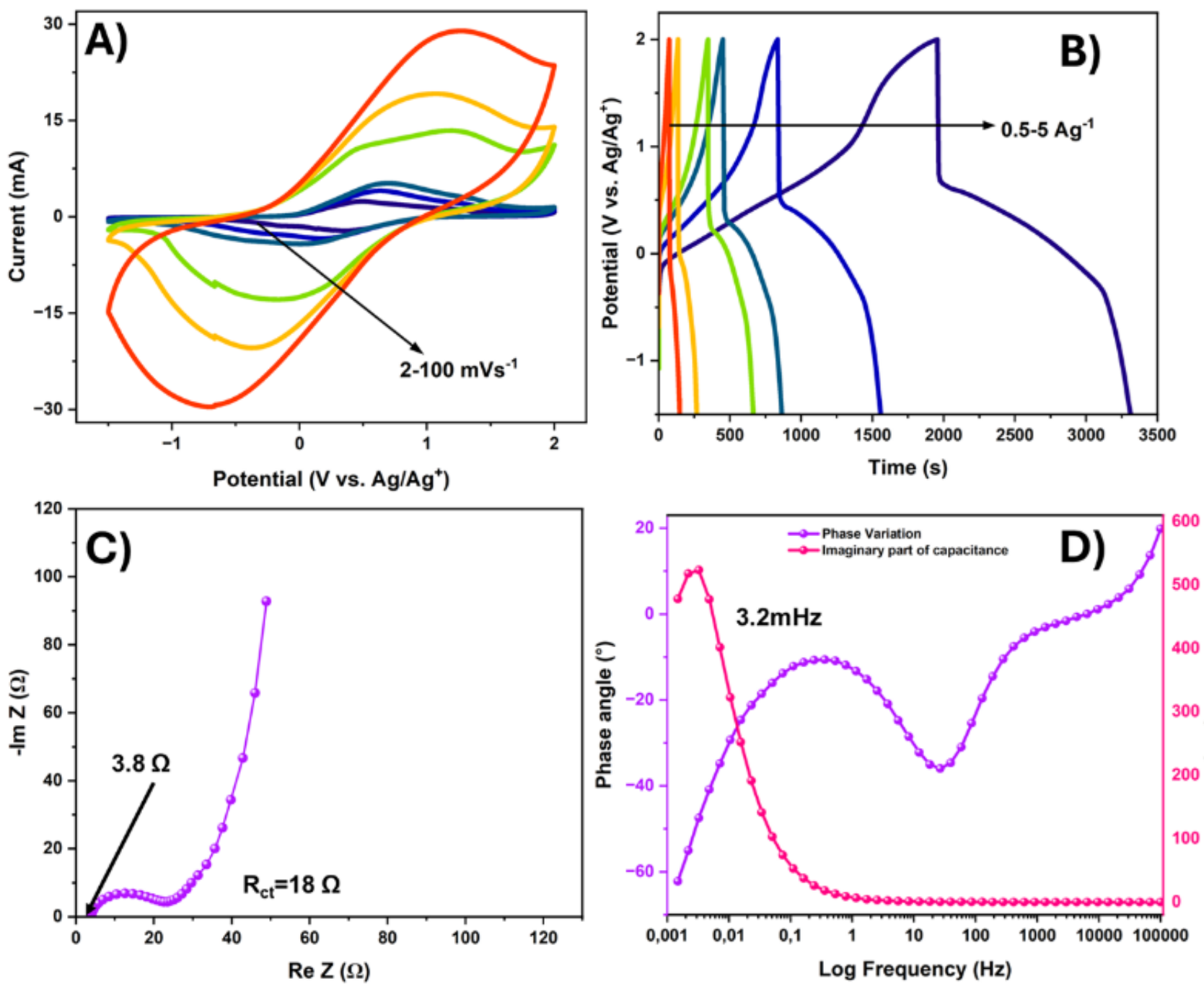

Fig. S6 (A) CV curves at various scan rates; (B) GCD curves at different current densities; (C) Nyquist plot; (D) Bode plot for PANI-PMP composite for 3-electrodes configuration.

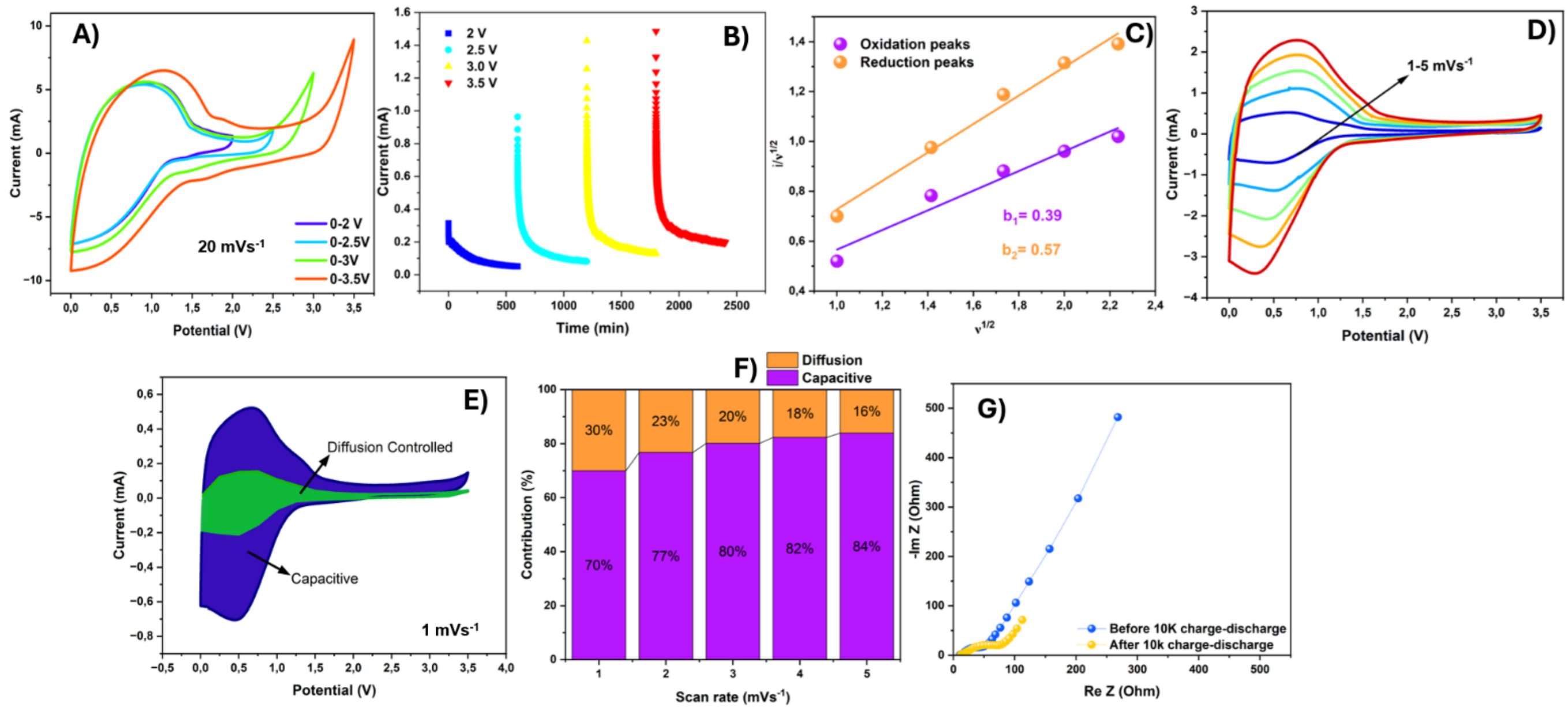

Fig. S7 (A) CV curves at different potential windows at 20 mVs^{-1}; (B) Chronoamperometric response at constant cell voltages; (C) Plot of log i vs. log *v*; (D) CV curves at various scan rates (E) CV response of capacitive and diffusion-controlled distribution; (F) Normalized ratios of capacitive and diffusion-controlled contributions; (G) Nyquist plot before and after 10K cycles of charge-discharge for symmetric cell.

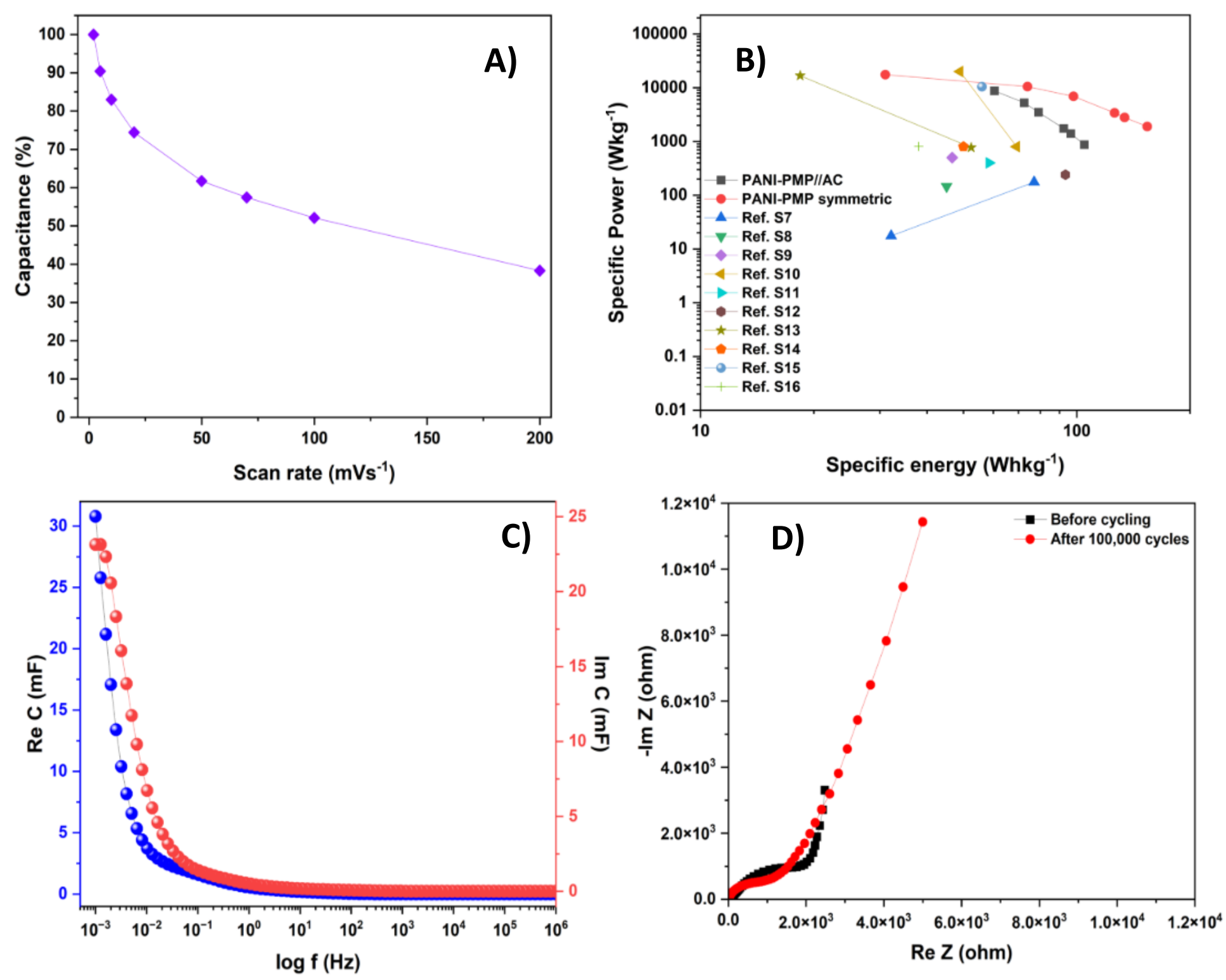

Fig. S8 (A) Specific capacities at various scan rates; (B) Extended Ragone plot comparing carbon-, metal oxide-, conducting polymer-, and MXene-based two-electrode cells; (C) Variation of the real and imaginary parts of the capacities with frequency; (D) Nyquist plot before and after 100 K cycles of charge-discharge for PANI-PMP//AC cell

 This journal is © The Royal Society of Chemistry 20xx

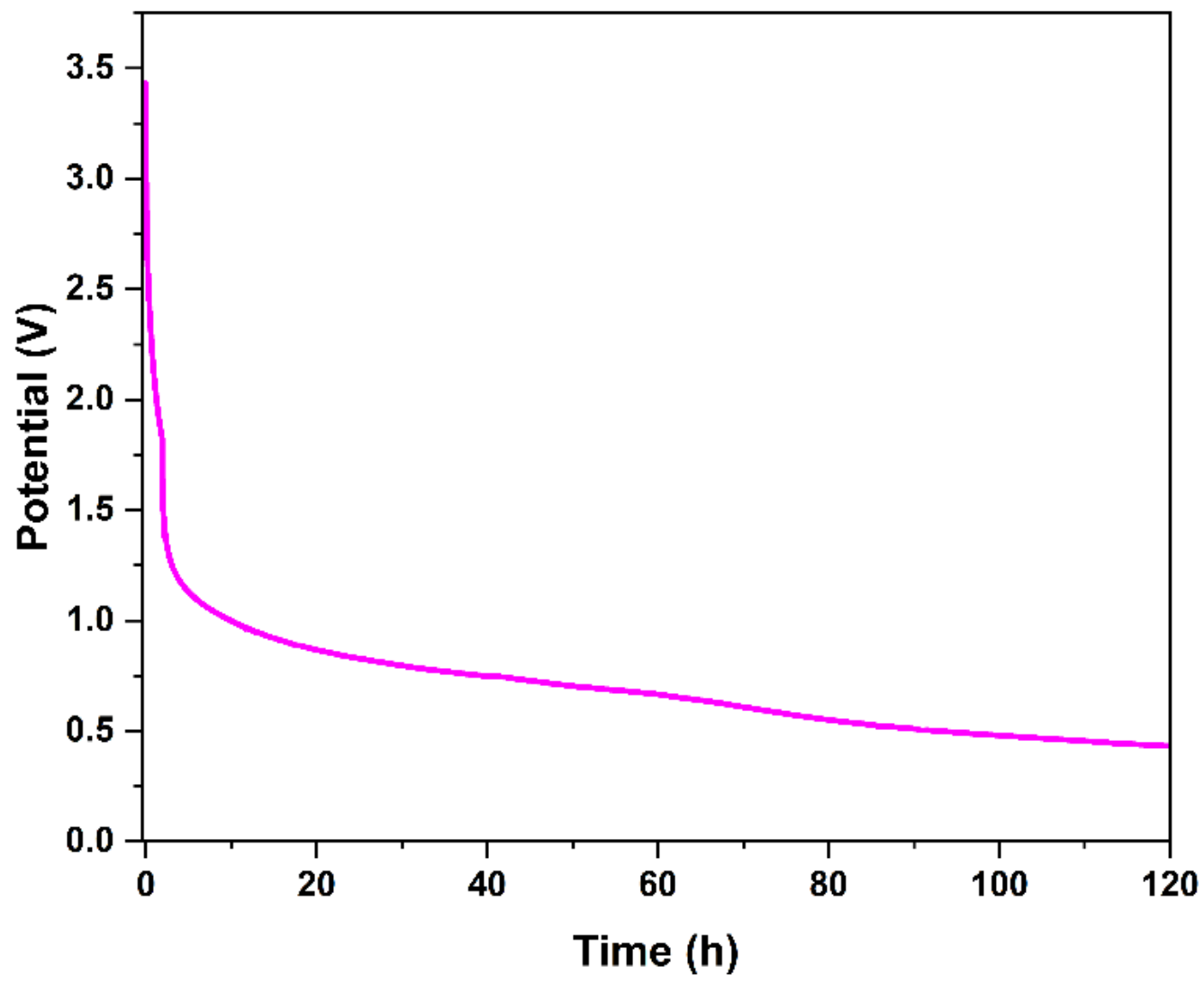

Fig. S9 Extended self-discharge analysis of the PANI–PMP//AC cell for120 h

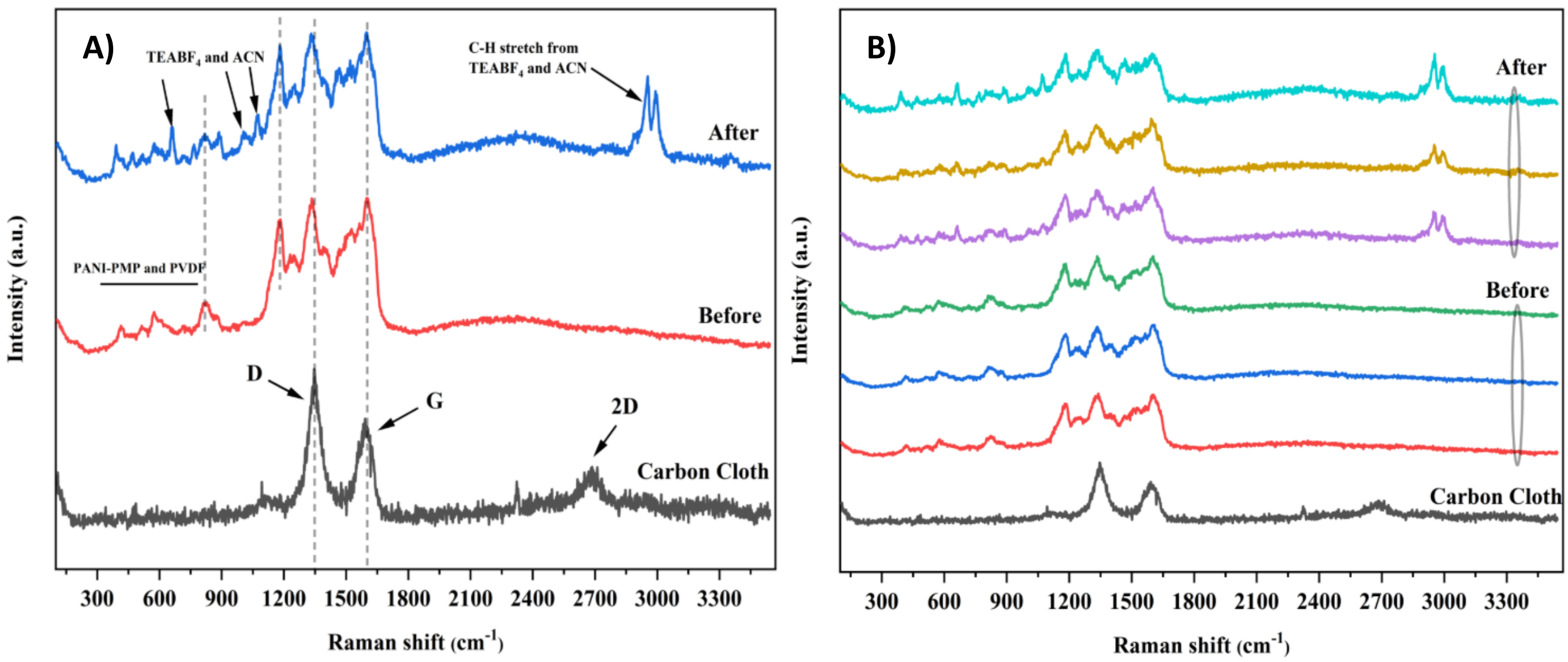

Fig. S10 (A) Raman spectroscopy of carbon cloth, the PANI–PMP structure before cycling, and after cycling, (B) Averaged Spectra

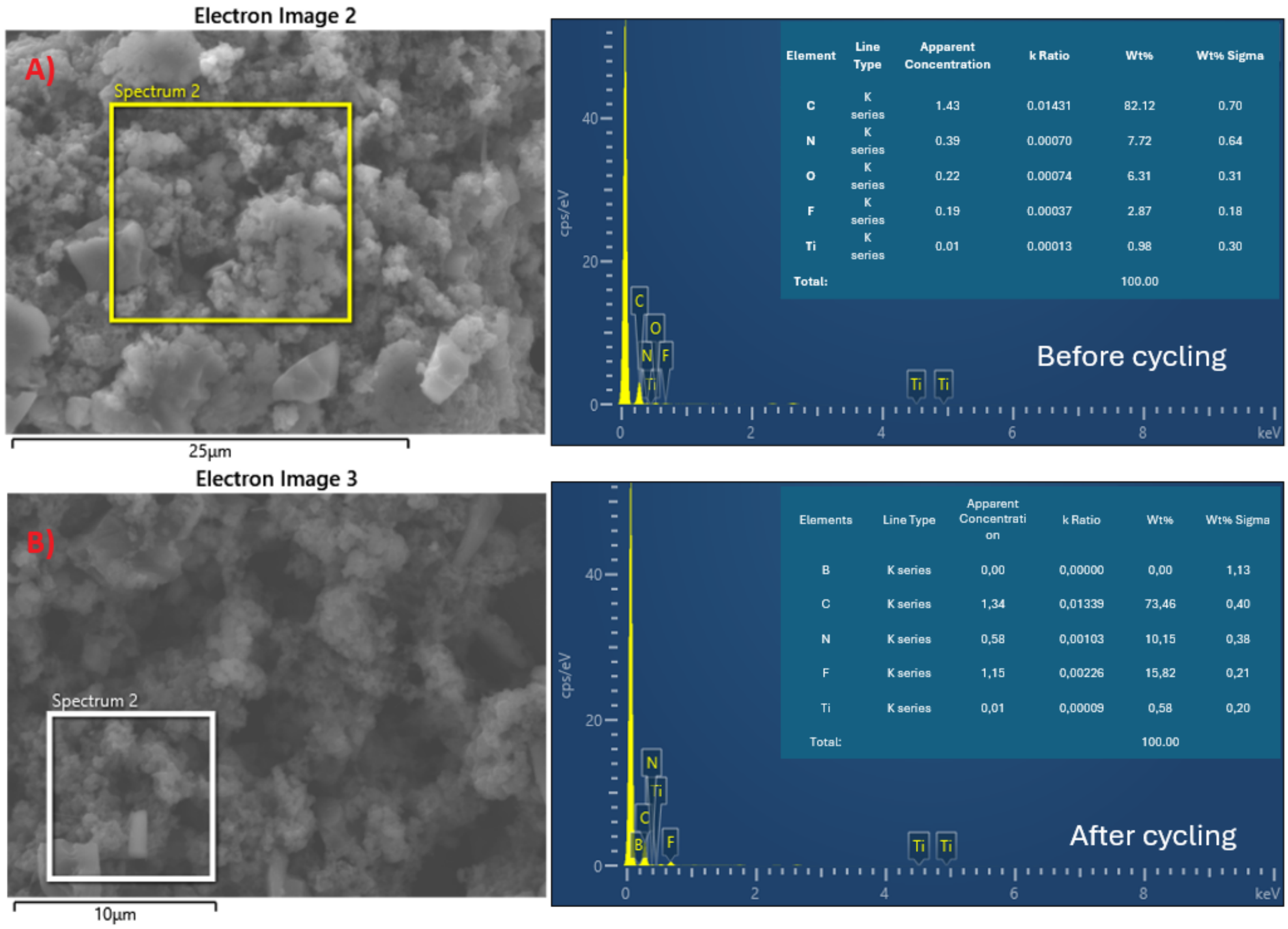

Fig. S11 EDS analysis of the PANI-PMP structure (A) before cycling, and (B) after cycling

List of Tables:

Table S1 SSAs and corresponding pore volumes, in pristine PMPs and PANI-PMP composites

Material	Surface area (m2/g)	Pore volume (cm3/g)
PMP	98 m^2/g	0.19 cm^3/g
PANI-PMP (1:1)	23 m^2/g	0.12 cm^3/g
PANI-PMP (2:1)	25 m^2/g	0.13 cm^3/g

This journal is © The Royal Society of Chemistry 20xx

Please do not adjust margins

Table S2 XPS peak fitting results of the regions: Ti 2p, C 1s, O 1s, and N 1s for PMP and PANI:PMP 1:1 cold pressed disc.

Region	Sample	BE [eV][a]	FWHM [eV][a]	Fraction	Assigned to	Reference
Ti 2p	PMP	459.3 (465.0)	1.1 (2.1)	1.0	Ti^{+4}	1
	PANI:PMP 1:1	459.4 (465.1)	1.1 (2.1)	1.0	Ti^{+4}	1
C 1s	PMP	284.7	1.4	0.07	C-H	2
		285.8	1.4	0.38	C-O	2
		287.3	1.4	0.48	C=O	2
		289.0	1.4	0.05	COOH/COOR	2
		289.7	1.4	0.02	pi	2
	PANI:PMP 1:1	284.8	1.0	0.35	C-H	2
		285.6	1.0	0.30	C-N	2
		286.3	1.0	0.18	C-O	2
		287.1	1.0	0.09	C=O	2
		288.1	1.0	0.06	COOH/COOR	2
		289.2	1.0	0.02	pi	2
O 1s	PMP	530.8	1.2	0.84	TiO_2	1
		531.2	1.6	0.11	Carboxylic groups	3
		533.0	2.0	0.05	$H_2O_{ads.}$	4
	PANI:PMP 1:1	530.7	1.0	0.25	TiO_2	1
		532.1	2.2	0.60	Carboxylic groups	3
		533.9	2.0	0.15	$H_2O_{ads.}$	
N 1s	PMP	400.6	2.0	0.21	Ammonium $(NH_4)^{+1}$	5
		403.5	1.3	0.79	from TMAOH during synthesis	
	PANI:PMP 1:1	399.9	1.5	0.75	Polyaniline	6
		401.4	2.0	0.25	Ammonium $(NH_4)^{+1}$ from TMAOH	5

This journal is © The Royal Society of Chemistry 20xx

Please do not adjust margins

Please do not adjust margins

Table S3 The value of specific capacities and corresponding energy densities from CV at different scan rates:

Scan rate (mVs^{-1})	Specific Capacity (Cg^{-1})	Specific Energy ($Whkg^{-1}$)
2	436	212
5	340	165
10	290	141
20	223	108
50	128	62
70	102	49
100	77	37
200	44	21

Table S4 The value of specific capacities, coulombic efficiency and corresponding energy and power densities from GCD at different current densities:

Current density (Ag^{-1})	Specific Capacity (Cg^{-1})	Specific Energy ($Whkg^{-1}$)	Specific Power ($kWkg^{-1}$)	Coulombic efficiency (%)
0.5	290	154	1.9	95
0.8	276	134	2.8	98
1	259	126	3.4	99
2	202	98	6.9	100
3	153	74	10.5	100
5	65	31	17.5	100

This journal is © The Royal Society of Chemistry 20xx

Please do not adjust margins

Please do not adjust margins

Table S5 The value of components in the equivalent series circuit for the PANI-PMP symmetric cell:

**Z fit Analysis**<br>Equivalent circuit: R1+C1/(R2+Q2/(R3+W3))+C4/R4	
R1	10,66 Ohm
C1	0,249 1e-3 F
R2	4,875 Ohm
Q2	0,094 68 F.s^(a - 1)
R3	1 786 Ohm
W3	39,4e48 Ohm.s^-1/2
C4	7,96e-3 F
R4	26,23 Ohm

Table S6 Literature for for Carbon-, Metal oxide-, conducting polymer-, and MXene-based two-electrode SCs:

Category	Electrode / Device	Specific Energy (Wh kg^{-1})	Specific Power (W kg^{-1})	Device configuration	References
Carbon Based	PHPCs-4 // PHPCs-4	77.0	175	Symmetric; ionic-liquid electrolyte, 3.5 V	[7]
Carbon Based	Napier-grass activated carbon // activated carbon	45.1	145.23	Symmetric	[8]
Carbon Based	AS-800 // AS-800	46.67	500	Symmetric	[9]
Metal oxide	$NiCo_2O_4$/rGO/NF // Activated Carbon	69.2<br>48.9	800<br>20,000	Asymmetric	[10]
Metal oxide	$CoMoO_4$@Co_3O_4 // Activated Carbon	58.5	400	Asymmetric, ~1.7 V	[11]
Conducting polymer	MoS_2/MWCNT/PPy // Activated Carbon	93.33	240.17	Asymmetric	[12]
Conducting polymer	PEDOT:PSS/NCNF // PEDOT:PSS/NCNF	52.4<br>18.4	771.7<br>16,800	Symmetric	[13]
Conducting polymer	WS_2/PANI // WS_2/PANI	50.0	800	Symmetric	[14]
MXene	$Ti_3C_2T_x$/Mesoporous Carbon ASC	56.0	10,423	Coin-cell asymmetric device	[15]
MXene	$Ti_3C_2T_x$/PANI // $Ti_3C_2T_x$/PANI	38.0	808	Symmetric	[16]

This journal is © The Royal Society of Chemistry 20xx

Please do not adjust margins

Please do not adjust margins

Table S7 The value of components in the equivalent series circuit for PANI-PMP//AC asymmetric cell:

**Z fit Analysis**<br>Equivalent circuit: R1+C2/R2+Q3/R3+C4/R4	
R1	11.63 Ohm
C2	0.049 86 F
R2	59 041 Ohm
Q3	1.967e-3 F.s^(a - 1); a3 = 0.775 3
R3	2 254 Ohm
C4	1.35e-3 F
R4	130.3 Ohm

List of Equations:

Galvanostatic capacitance: $C = \frac{\int iVdt}{\int_{V_i}^{V_f} VdV} = \frac{2i_m \int Vdt}{V^2 \left\{ \begin{smallmatrix} V_f \\ V_i \end{smallmatrix} \right.}$ F ………… (S1)

For Linear response: C= IΔt/mΔV F/g

Specific capacity (Q/m) = $\frac{1}{m}\int_{t1}^{t2} I(t)dt$ C/g

Energy density: $E = 1/m\int V(t)I(t)dt$ Wh/kg ………… (S2)

Power density: $P = (1/m\int V(t)I(t)dt)/t$ W/kg ………… (S3)

where *I, m,* dt and *V* are the discharge current, the mass of the single electrode, time, and potential window, respectively. In the case of a symmetric device, m is the mass of both electrodes (active material). For three-electrode measurements, the specific capacity is normalized to the mass of the active PANI-PMP material in the working electrode (80% of the total weight). For the symmetric two-electrode cell, the reported device-level quantities are normalized to the combined active-material mass of both electrodes. For the asymmetric PANI-PMP//AC device, the combined active masses of the positive and negative electrodes are used.

 This journal is © The Royal Society of Chemistry 20xx

Please do not adjust margins

Please do not adjust margins

The total impedance is given by,

$$Z(\omega) = Z'(\omega) + jZ"(\omega), ...(S4),$$

where $\omega = 2\pi f$, f is the frequency of the input ac signal.

The Total capacitance is given as,

$$c(\omega) = c'(\omega) - jc"(\omega)................. (S5),$$

where real the part of the capacitance $c'(\omega) = \frac{Z"(\omega)}{\omega|Z(\omega)|^2}$ .... (S6) and

the imaginary part of the capacitance $c"(\omega) = \frac{Z'(\omega)}{\omega|Z(\omega)|^2}$ .... (S7)

References:

1 U. Diebold and T. E. Madey, *Surface Science Spectra*, 1996, **4**, 227–231.
2 X. Chen, Wang ,Xiaohui and D. and Fang, *Fullerenes, Nanotubes and Carbon Nanostructures*, 2020, **28**, 1048–1058.
3 G. P. López, D. G. Castner and B. D. Ratner, *Surface and Interface Analysis*, 1991, **17**, 267–272.
4 S. Yamamoto, H. Bluhm, K. Andersson, G. Ketteler, H. Ogasawara, M. Salmeron and A. Nilsson, *J. Phys.: Condens. Matter*, 2008, **20**, 184025.
5 A. Mohtasebi, T. Chowdhury, L. H. H. Hsu, M. C. Biesinger and P. Kruse, *J. Phys. Chem. C*, 2016, **120**, 29248–29263.
6 S. Golczak, A. Kanciurzewska, M. Fahlman, K. Langer and J. J. Langer, *Solid State Ionics*, 2008, **179**, 2234–2239.
7 K. Wang, X. Dong, R. Yan, S. Jia, B. Cui, Q. Zhao and Y. Song, *Electrochimica Acta*, 2024, **504**, 144962.
8 E. Taer, A. Agustino, A. Fudholi, N. Chitraningrum and R. Taslim, *Diamond and Related Materials*, 2025, **155**, 112380.
9 E. Elanthamilan and S.-F. Wang, *Journal of Energy Storage*, 2025, **132**, 117738.
10 Z. Wei, Q. Wang, M. Qu and H. Zhang, *ACS Appl. Mater. Interfaces*, 2024, **16**, 18734–18744.
11 J. Li, H. Lin, Z. Ai, H. Feng, Y. Li, J. Xu and H. Xie, *Journal of Energy Storage*, 2025, **107**, 115041.
12 N. Jhanjhariya and S. Lata, *Journal of Energy Storage*, 2024, **82**, 110577.
13 M. Panapoy and B. Ksapabutr, *Journal of Energy Storage*, 2024, **94**, 112285.
14 V. V. Mohan and R. B. Rakhi, *Electrochimica Acta*, 2024, **498**, 144657.
15 S. D. Sutar and A. Swami, *Nanoscale*, 2025, **17**, 4750–4764.
16 S. M. Varghese, V. V. Mohan, S. Suresh, E. Bhoje Gowd and R. B. Rakhi, *Journal of Alloys and Compounds*, 2024, **973**, 172923.

This journal is © The Royal Society of Chemistry 20xx

Please do not adjust margins
